# Constraint-based modelling of metabolic dysregulation in Gaucher disease: mitochondrial dysfunction and disrupted cholesterol homeostasis

**DOI:** 10.1186/s13023-026-04206-8

**Published:** 2026-01-20

**Authors:** Yanjun Liu, Xi Luo, Samira Ranjbar, Johannes M. F. G. Aerts, Martijn van der Lienden, Andrea Dardis, Ronan M. T. Fleming

**Affiliations:** 1 School of Medicine, University of Galway, University Road, Galway, Ireland; 2 Digital Metabolic Twin Centre, University of Galway, Galway, Ireland; 3 Department of Medical Biochemistry, Leiden Institute of Chemistry, Leiden University, Leiden, Netherlands; 4 Regional Coordinator Centre for Rare Diseases, University Hospital of Udine, Udine, Italy

**Keywords:** Gaucher disease, Lipid metabolism, Cholesterol homeostasis, Metabolic modelling, Constraint-based modelling

## Abstract

**Background:**

Gaucher disease (GD) is a lysosomal storage disorder caused by mutations in the *GBA1* gene, leading to deficient glucocerebrosidase activity and accumulation of glucosylceramide in macrophages. Beyond lysosomal dysfunction, GD is associated with widespread metabolic abnormalities, yet the molecular basis of these changes remains incompletely understood. This study employed constraint-based genome-scale metabolic modelling to investigate systemic metabolic reprogramming in GD macrophages, aiming to uncover disrupted pathways and mechanistic drivers of disease phenotypes.

**Results:**

A total of 150 pairs of high-quality macrophage-specific models under Gaucher and control conditions were developed using a semi-automated pipeline to integrate transcriptomic, exometabolomic and bibliomic data. These Gaucher models captured disease-specific perturbations by incorporating gene expression profiles from GD macrophages. Simulations predicted a shift from oxidative phosphorylation to glycolysis under energy stress in GD, attributed to impaired mitochondrial ATP transport and reduced activity of respiratory complexes. Lipid metabolism was profoundly altered, with increased *de novo* ceramide synthesis, defective ganglioside processing, and dysregulated cholesterol metabolism. Despite clinically observed hypocholesterolaemia, the models predicted upregulated intracellular cholesterol biosynthesis, suggesting a disconnect between intracellular and systemic cholesterol pools. Reporter metabolite analysis further highlighted cholesterol, sphingolipids, and acylcarnitine as hubs of transcriptional dysregulation. Robust metabolic transformation analysis predicted *ASAH1*(acid ceramidase) and *CPT1A* (carnitine palmitoyl transferase 1 A) as potential modifier genes influencing lipid catabolism and mitochondrial function. Several model predictions were corroborated by independent experimental findings, supporting their biological plausibility.

**Conclusions:**

This study demonstrates the utility of constraint-based metabolic modelling in elucidating the systems-level metabolic dysfunction underlying GD. The results highlight a core axis of mitochondrial and lipid metabolic disruption, particularly involving cholesterol homeostasis, as a central feature of disease pathophysiology. These predictions provide mechanistic insight into the cellular consequences of lysosomal dysfunction and identify candidate metabolic biomarkers and therapeutic targets. The modelling framework developed here supports hypothesis generation and future applications in precision medicine for lysosomal storage disorders.

**Graphical Abstract:**

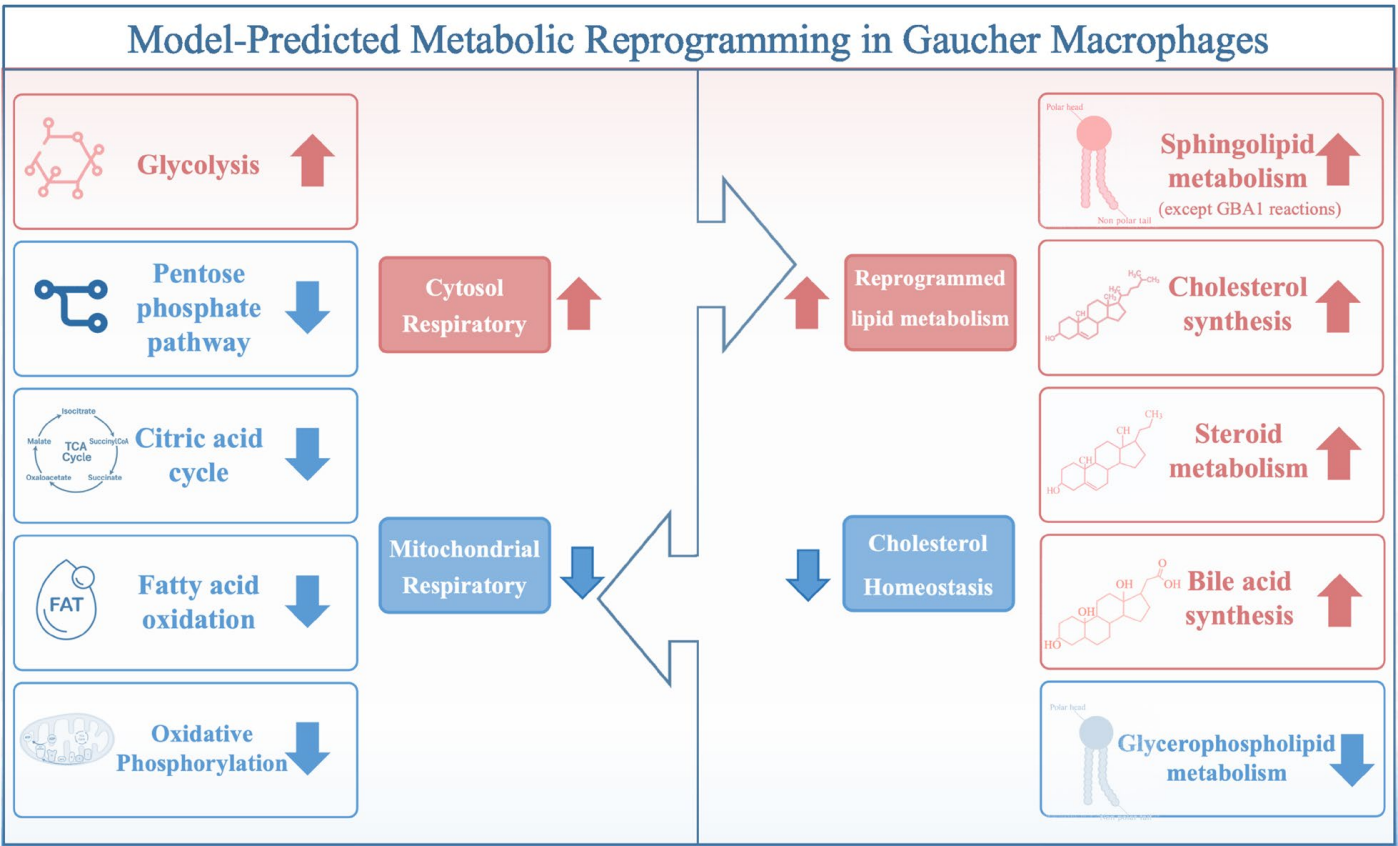

**Supplementary Information:**

The online version contains supplementary material available at 10.1186/s13023-026-04206-8.

## Background

Lysosomes degrade macromolecules from autophagy and endocytosis to maintain metabolic homeostasis. Genetic defects in lysosomal enzymes impair this balance, causing lysosomal storage disorders marked by substrate accumulation [1]. Gaucher disease (GD) represents a prototypical example, caused by biallelic *GBA1* mutations that lead to β-glucocerebrosidase deficiency and result in lysosomal accumulation of glucosylceramide [2]. Macrophages, which clear glycosphingolipid-rich membranes from senescent blood cells, are particularly affected and differentiate into lipid-laden Gaucher cells [3]. Clinically, GD patients commonly experience symptoms such as hepatosplenomegaly, cytopenia, and skeletal pathology. In certain cases, neurological system involvement emerges, marked by manifestations such as epilepsy, oculomotor abnormalities (ocular apraxia) and progressive motor impairment. GD is typically categorised into three subtypes – type I (the non-neuronopathic variant, marked by visceral involvement without neurological features), type II (the acute neuronopathic form), type III (the subacute neuronopathic form) [4] 1.

Although genotype contributes to disease heterogeneity, *GBA1* variants alone do not fully predict clinical severity, implicating additional genetic and metabolic modifiers. Altered expression of genes involved in lysosomal trafficking (*SCARB2* [7]), lysosomal activation of glucocerebrosidase (*PSAP* [7]), sphingolipid synthesis (*UGCG* [8]), endoplasmic reticulum-Golgi protein trafficking (*CLN8* [9]), neuronal excitability (*GRIN2B* [10]), and lysosomal integrity and inflammation (*PGRN* [11–13]) has been associated with variation in GD severity 2. Beyond genetics, systemic metabolic stress such as hypermetabolism, insulin resistance and malnutrition, also influences disease expression [14–21]. Malnutrition is common in untreated children, while adults on enzyme replacement therapy may gain weight due to reduced energy expenditure [16, 20]. Vitamin D deficiency is also widespread, contributing to skeletal and immune complications [16]. These findings underscore the idea that GD is not solely a lysosomal disorder but also involves broader disruptions in metabolic homeostasis.

Animal models have illuminated GD mechanisms, though each has limitations. Chemically-induced [22, 23] and genetic-knockout mice [24] are common, but Chemically-induced mice does not replicate endoplasmic reticulum stress and full knockouts are lethal [25–28]. Conditional knockouts allow tissue-specific study but are technically complex and costly [29–31]. Zebrafish [32–34] and drosophila [35–40] models offer insight into glucocerebrosidase function and therapeutic screening, though their physiology differs significantly from humans. A further limitation common to these systems is that they do not fully capture the behaviour of the cell type most critically involved in GD pathogenesis: the macrophage [3]. Macrophages are the primary site of pathological lipid accumulation and are key drivers of inflammation and biomarker production [3]. However, human macrophage models of GD remain scarce, and most available patient-derived cellular models are fibroblasts, which, although accessible, do not recapitulate macrophage-specific lysosomal and metabolic functions.

Computational modelling offers a complementary strategy to overcome these challenges by providing a systematic, mechanistic representation of human cellular metabolism. Genome-scale metabolic models integrate curated biochemical knowledge into a comprehensive network of metabolic reactions [41]. Leveraging the holistic nature of network structures, genome-scale metabolic models enable systems-level investigation of GD by simulating genetic or environmental perturbations [41–44]. Unlike in vivo models, in silico simulations allow safe and flexible testing of gene deletions and therapeutic targets, even when knockouts are lethal [31]. Integration of transcriptomic, proteomic, and metabolomic data enables the construction of context-specific models that better reflect macrophage physiology [45, 46]. This enables researchers to explore tissue-specific metabolism, identify biomarkers, and simulate therapeutic interventions under varying physiological conditions [47, 48]. Additionally, in silico approaches avoid species-specific discrepancies in lipid metabolism, immune function, and gene regulation, which often limit the translational relevance of animal models [49, 50].

Here, we construct macrophage-specific genome-scale metabolic models representing healthy and *GBA1*-deficient states by integrating transcriptomic, metabolomic, and literature-curated data. Using constraint-based modelling techniques, we investigate how lysosomal dysfunction reshapes global metabolic adaptations in macrophages. This framework provides a mechanistic basis for interpreting metabolic adaptation in GD and identifies candidate pathways, biomarkers, and metabolic vulnerabilities relevant to disease progression and therapeutic development.

## Method

**Fig. 1:**
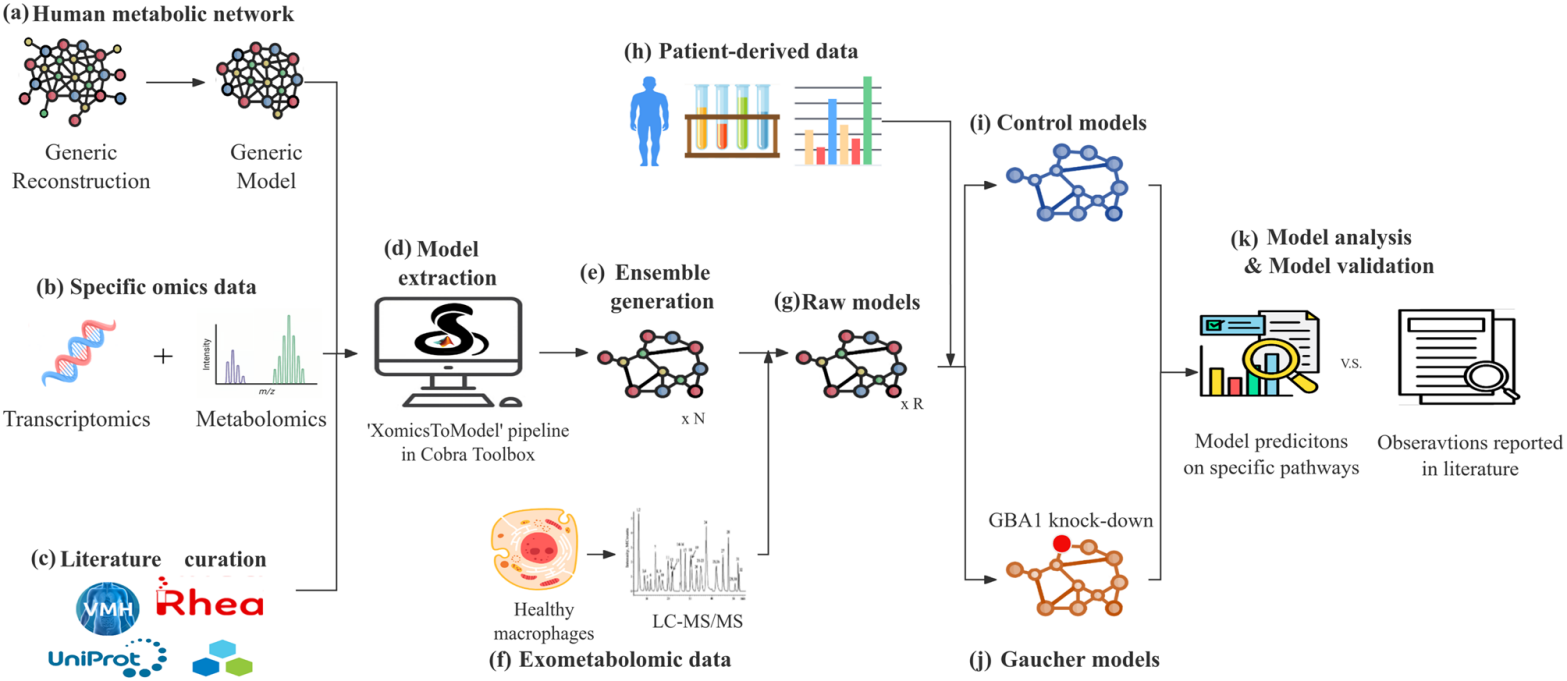
Overview of model generation, evaluation, and validation. (**a**) The generic human metabolic reconstruction, Recon3D [41], was converted into a computational model. (**b**–**c**) Omics data—including transcriptomic and metabolomic profiles from healthy macrophages, as well as bibliomic data from literature curation—were used to identify macrophage-relevant genes and metabolites. (**d**) The ’XomicsToModel’ pipeline [46], was then applied to extract a macrophage-specific metabolic network by integrating these multi-omics data with the generic model. (**e**) By varying input parameters, N draft models were generated. (**f**) These draft models were validated by comparing model predictions against experimentally measured uptake and secretion rates in macrophages. (**g**) The validated models served as a foundation to generate (**i**) Control models and (**j**) Gaucher models by (**h**) incorporating gene expression data from Gaucher patients and simulating GBA1 knockdown. (**k**) Comparative analysis of the Control and Gaucher models enabled prediction of metabolic alterations specific to Gaucher macrophages

An ensemble of computational models was developed to capture the metabolic alterations in macrophages associated with GD. An overview of the model development, evaluation, and simulation framework is shown in Fig. 1.

### Model development

To generate macrophage-specific metabolic models, we identified a core set of genes and reactions expected to be active in macrophages, supported by transcriptomic, proteomic, and bibliomic data. We then applied the established ‘XomicsToModel’ pipeline [46] to map the core list onto ‘Recon3D’ [41], the most comprehensive human metabolic reconstruction. This enabled us to extract a context-specific subnetwork that maximised the inclusion of core macrophage-associated reactions (Fig. 1, step a - d).

#### Transcriptomic data

A public transcriptomic dataset of macrophages differentiated from peripheral blood monocytes of healthy donors was used (GSE185919) [51]. Cells were isolated using CD14 + Microbeads with > 95% purity and incubated in DMEM medium supplemented with 10% foetal bovine serum. Gene expression was quantified as Fragments Per Kilobase of transcript per Million mapped reads (FPKM), and actively expressed genes were defined using a threshold of FPKM > 1*10^1^ under these culture conditions.

#### Bibliomic data

To reduce bias from specific culture conditions, a macrophage-specific metabolic network was curated using multiple biochemical and proteomic resources. Evidence was gathered from peer-reviewed macrophage literature, and public databases including the Human Protein Atlas [52], the Transporter Classification Database [53]. A gene was included when any macrophage-specific experimental source supported its activity, such as transcript abundance above the defined threshold (FPKM > 1*10^1^), PCR detection, protein-level confirmation, or reported enzymatic activity. Reactions were incorporated when enzymatic function in macrophages had been demonstrated through biochemical assays, immunostaining, or curated database annotations. Pathways essential for macrophage biology, such as central carbon metabolism, sphingolipid metabolism, and biomass-related processes, were systematically reviewed to ensure biologically consistent coverage.

#### Generic metabolic network of human metabolism

The comprehensive metabolic network of human metabolism, ‘Recon3D’ [41], served as the foundation to extract the macrophage-specific metabolic network. The list of core genes and reactions identified through transcriptomics and bibliomic data was mapped to the ‘Recon3D’ network [41] based on the gene-protein-reaction rule, which states that genes encode proteins, some of which are metabolic enzymes capable of catalysing reactions. In this context, the presence of genes supports the existence of the corresponding reactions in the network.

#### Model extraction

The generic network was integrated macrophage-specific gene and reaction lists using the ‘XomicsToModel’ pipeline [46] to extract a cell type-specific subnetwork representing macrophage metabolism. This pipeline prioritises the inclusion of core genes and reactions while enforcing stoichiometric consistency, flux consistency, and thermodynamic feasibility. These checks ensure compliance with biochemical and physical principles, such as mass balance, and confirm that every retained reaction can carry flux and satisfies thermodynamic constraints [54]. The pipeline also incorporated several user-defined parameters at the data mapping step, the constraining step, and during solver selection for model consistency checks. Different parameter combinations were tested during model development (S Table 1, S File 1).

The models were formulated by constructing a stoichiometric matrix from reaction coefficients and defining fluxes as a vector. Reaction bounds reflecting reaction capacity were defined using quantitative constraints based on medium composition and literature-derived biological requirements (S File 2), following an established approach [55].

### Model evaluation

Draft models of healthy macrophages were further refined to optimise their predictive accuracy against experimentally measured exometabolomic data [56] (Fig. 1, step e - g). Simulated nutrient uptake and secretion fluxes were compared with experimentally measured exchange rates through a series of consistency checks. Models that best reproduced the metabolic profiles of cultured healthy macrophages were retained as representative controls. Quality control tests [57] were also performed to ensure that these models could replicate key physiological macrophage functions.

#### Model prediction

The exchange rates of metabolites were predicted using flux balance analysis (FBA) [42] and entropic flux balance analysis (EntropicFBA) [55, 58]. Detailed mathematical formulations for both methods are provided in the Supplementary Information (S File 1).

#### Consistency check between simulated and measured exchange rates

To evaluate model performance, a dataset of non-targeted exometabolomic profiling of in vitro cultured macrophages [56] was used to compare simulated nutrient uptake and secretion fluxes with experimentally measured exchange rates. The dataset reports exchange rates for metabolites that were measurable under the experimental conditions. Fourteen metabolites were identified as taken up by the cells and forty as secreted (S File 3). Only these metabolites were used for model validation and selection.

Model predictions were then assessed using a multi-level validation framework encompassing qualitative (correct predictions/total Predictions), semi-quantitative (Spearman’s rank correlation coefficient), and quantitative (weighted Euclidean distance) consistency checks (S Table 2). Models that best reproduced the exometabolomic profiles across these evaluation criteria were selected to represent the healthy macrophage state.

### Simulation of gaucher metabolism

Metabolic alterations in GD were simulated using validated healthy macrophage models as a reference. While *GBA1* deficiency was first simulated through single gene knockout to examine its direct metabolic consequences, this approach alone could not capture the broader systemic effects associated with the disease, such as endoplasmic reticulum stress and chronic inflammation. To better reflect these secondary alterations and transcriptional adaptations, transcriptomic data from two independent GD macrophage studies [59, 60] were integrated to construct condition-specific metabolic models of *GBA1*-deficient macrophages and *GBA1*-normal macrophages (referred to as GD models and control models throughout the text). This enabled simulation of transcriptionally driven metabolic reprogramming under disease conditions (Fig. 1, step g-k).

#### Data-driven customisation of metabolic models for gaucher disease

Two transcriptomic datasets (GSE183484, GSE13675) [59, 60] were used to identify the differentially expressed genes in GD. GD models were generated by adjusting the bounds of GBA1-catalysed reactions and reactions associated with the differentially expressed genes in validated models of healthy macrophages. Reaction bounds were widened for upregulated genes and tightened for downregulated genes, using a qualitative scaling scheme designed to capture relative regulatory trends while maintaining model feasibility. This approach avoids unrealistic assumptions, as transcript levels are not directly proportional to enzyme abundance or catalytic capacity.

Because quantitative enzyme capacities are unavailable for most reactions and direct scaling based on expression fold changes often yields infeasible models, a qualitative strategy was adopted that preserves only the direction of regulation rather than imposing absolute flux values. The upper bound of the reaction catalysed by *GBA1* was reduced to 10% of its reference value to reflect severe loss of glucocerebrosidase activity [4] .Upper bounds for reactions associated with downregulated genes were reduced to 50%, limiting their maximal flux capacities, while lower bounds for reactions associated with upregulated genes were increased to 150%, expanding their minimal feasible fluxes. These moderate adjustments were introduced within the entropicFBA framework to maintain thermodynamic and mass balance consistency. Control and GD model instances were paired for comparison, and model filtering was performed using t distributed stochastic neighbour embedding of predicted fluxes to retain instances that showed appropriate clustering according to the applied perturbation.

To address the concern regarding the arbitrariness of these scaling choices, we performed a robustness analysis using alternative parameter ranges (30% to 70% for down regulated reactions and 130% to 170% for up regulated reactions).

To ensure meaningful comparisons, model instances were filtered via t-distributed stochastic neighbour embedding (t-SNE) on predicted fluxes, assessing whether models clustered by intervention type and qualitatively reflected gene expression-driven differences.

#### Strategies for functional annotation and regulatory inference in GD models

The analyses of models followed a hierarchical structure: differential flux analysis identified altered reactions in GD; reaction correlations and transcription factor enrichment characterised their network-level coordination; reporter metabolite analysis integrated transcriptomic signals to highlight candidate metabolic biomarkers; and metabolic transformation analysis prioritised gene perturbations capable of shifting the GD flux state.

##### Simulation setup

To promote biologically meaningful flux through the metabolic network, several key reactions were included in the objective function. These comprised the ‘biomass_maintenance’ reaction, which accounts for the baseline molecular cost required to sustain basic cellular viability; ‘ATPM’, representing the energy demands associated with non-metabolic cellular functions; ‘ATPS4mi’, which reflects the activity of mitochondrial ATP synthase; and the initial reactions in the ganglioside synthesis pathway, included to drive flux through this disease-relevant biosynthetic route. The lower bounds for ‘ATPM’ and ‘biomass_maintenance’ were set to 44.8 umol/gDW/hr and 3.45 umol/gDW/hr, respectively, in accordance with experimentally derived estimates for minimal cellular viability [61]. To simulate lysosomal substrate availability, the ‘EX_gangliosides[l]’ reaction, which represents the direct phagocytosis of complex gangliosides, was constrained to a fixed uptake bounds spanning a wide range (0.1, 1, 5, 10, umol/gDW/hr). This range was chosen to capture biologically plausible levels of glycosphingolipid internalisation while enabling assessment of the robustness of downstream analyses to this assumption.

##### Identification of dysfunctional pathways in gaucher models

To investigate metabolic alterations in GD, flux distributions for both GD and control models were first predicted using entropicFBA. This thermodynamically consistent approach enabled the estimation of steady-state reaction fluxes that reflect realistic cellular behaviour under both healthy and disease conditions. To identify reactions with altered activity between GD and control models, differential flux analysis was performed. Reactions exhibiting statistically significant differences in flux were defined using two criteria: $\:{\mathrm{l}\mathrm{o}\mathrm{g}}_{2}\left(\frac{{v}_{GD}}{{v}_{control}}\right)>1$, and $\:p$ < 0.05.

##### Identification of disease-specific interactions and transcription factors potentially modulating metabolism

Spearman correlation analysis was conducted on the subset of reactions whose fluxes were previously found to differ significantly between GD and control models. Correlations were deemed GD-specific if strong in GD ($\:\left|\rho\:\right|\ge\:0.7$) but weak in controls ($\:\left|\rho\:\right|\le\:0.3$), potentially reflecting altered coordination or compensatory responses. Pairwise flux correlations were computed within each model, followed by hierarchical clustering based on the absolute correlation matrix. To assess regulatory drivers, genes associated with co-regulated reactions were analysed for transcription factor enrichment using ChEA3 [62].

##### Discovery of candidate biomarkers in GD models

To identify transcriptionally regulated metabolic hubs in GD, gene expression data from iPSC-derived macrophages were integrated into the metabolic network using the reporter metabolites algorithm [63]. This method maps differential gene expression onto a bipartite graph linking enzymes to metabolites and calculates a Z-score for each metabolite based on the aggregate transcriptional changes of its associated enzymes (Detailed mathematical explanation provided in S File 1). Scores are normalised against random gene sets to account for background variation. Metabolites with the highest Z scores, which reflect coordinated regulation of adjacent enzymes, are designated as reporter metabolites, indicating network regions subject to strong transcriptional regulation. Statistical significance was assessed via randomisation-based null distributions.

##### Inference of putative modifier genes in GD models

To identify potential gene targets capable of shifting the metabolic state of GD towards a healthy phenotype, we applied robust Metabolic Transformation Analysis (rMTA) [64, 65] to the flux distributions predicted from GD and control models. rMTA is a computational framework designed to systematically evaluate the impact of single-gene perturbations on the global metabolic network. Its objective is to prioritise perturbations that steer the disease-associated flux distribution towards a predefined reference state, in this case the flux profile of the control models, while maintaining metabolic feasibility. To account for potential bidirectional metabolic shifts, the analysis was conducted in both directions, shifting from GD to control and from control to GD. This approach enabled the identification of genes whose perturbation may either restore metabolic balance in GD (candidate therapeutic targets) or drive the system away from homeostasis (putative modifiers).

All code used in this study was implemented in MATLAB R2024b (MathWorks Inc.) and relied on the COBRA Toolbox 3.0 [43], including the ‘XomicsToModel’ [46] pipeline for model generation and the EntropicFBA algorithm for further analysis. The solvers, such as Gurobi (v9.1.2) and Mosek (v10.0.40), are all interfaced with the COBRA Toolbox 3.0 [43].

## Results

### Generation of metabolic models of healthy and gaucher macrophages

#### Generation and evaluation of models

A total of 384 draft models were generated, of which 150 satisfied the evaluation criteria and were retained as representative models of healthy macrophage metabolism (S Fig. 1). These models achieved an average qualitative prediction accuracy of 65% and a mean Spearman correlation coefficient above 0.56 when comparing simulated and measured exchange rates (S File 3). Prediction accuracy was higher for nutrient uptake, reaching up to 90%, whereas accuracy for secreted metabolites was lower, typically 40–50% (S File 3).

**Fig. 2:**
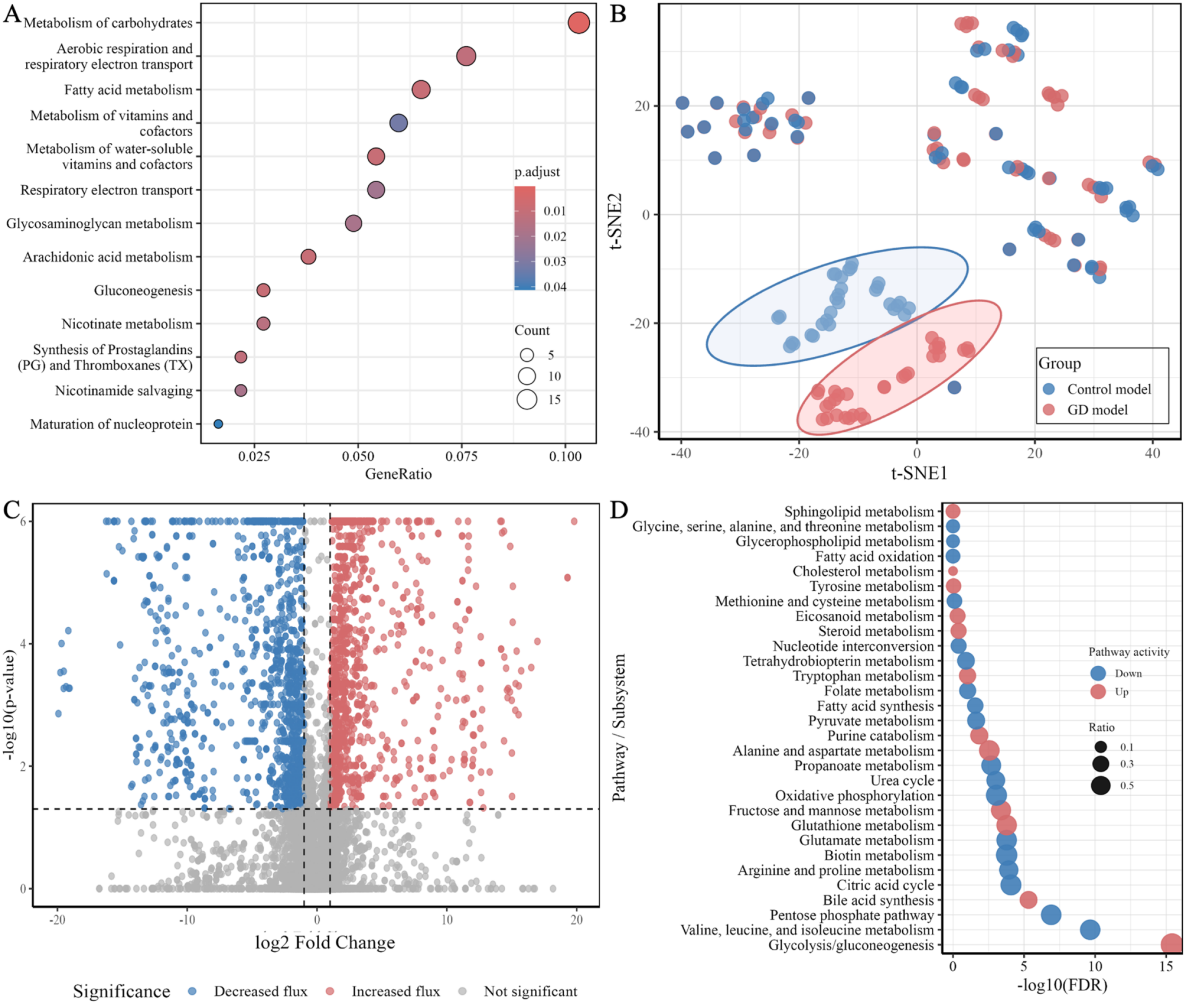
Transcriptomic and predicted fluxomic change in Gaucher disease. (**A**) Pathway enrichment analysis of differentially expressed metabolic genes identified from GD transcriptomic datasets. (**B**) t-SNE visualisation of flux distributions for GD and control models. Models with similar flux profiles cluster together, and two clearly separated clusters corresponding to GD and control states were identified. These clustered models were used to represent disease and healthy macrophages in subsequent analyses. (**C**) Volcano plot showing fold changes in reaction fluxes between GD and control models as predicted by EntropicFBA. (**D**) Pathway enrichment analysis of reactions with significantly altered fluxes, mapped to metabolic subsystems defined in Recon3D

Exometabolomic profiles predicted using EntropicFBA are shown (S Fig. 2)., together with the experimental measurements. Simulated uptake rates were generally consistent with the data, while secretion rates showed weaker agreement. The largest discrepancies occurred for essential amino acids. In the model, amino acids such as L-histidine, L-lysine, L-tryptophan, and L-phenylalanine were constrained to be taken up because they cannot be synthesised *de novo*. In contrast, the experimental data suggested net accumulation in the spent medium, likely reflecting contributions from serum components or cell-specific release mechanisms unrelated to de novo synthesis. Because these metabolites did not provide a biologically meaningful constraint for model selection, they were excluded from the decision criteria used to identify the final set of healthy macrophage models.

#### Simulation of gaucher metabolism

**Fig. 3:**
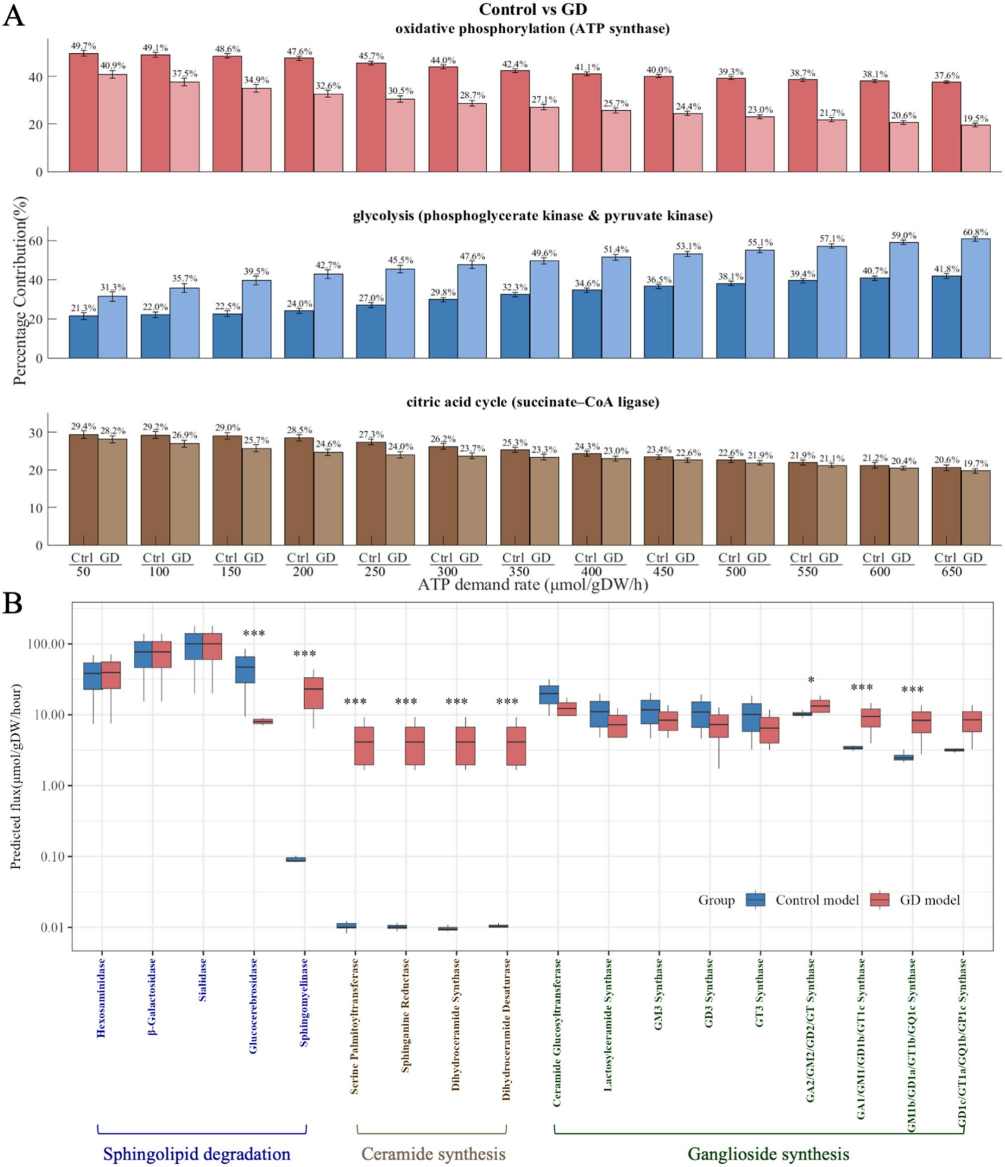
Metabolic pathway alterations in Gaucher disease. (**A**) Predicted ATP contribution from the three main energy-producing pathways. Relative contributions of glycolysis, the citric acid cycle and oxidative phosphorylation to total ATP production in control and GD models, estimated using entropicFBA. (**B**) Predicted activity of key enzymes in sphingolipid metabolism. Flux distributions for enzymes across the three major branches of sphingolipid metabolism in control and Gaucher disease models are shown on a log_10_ scale (umol/gDW/hour). X-tick labels are colour-coded to indicate pathway assignment: sphingolipid degradation (dark blue), de novo ceramide synthesis (brown) and complex ganglioside synthesis (dark green). Statistical significance is indicated as follows: *p* < 0.05 (*), *p* < 0.01 (**), and *p* < 0.001 (***)

Metabolic alterations associated with GD were simulated by mapping disease-specific gene expression onto the 150 validated models of healthy macrophage, which served as a reference. A total of 994 differentially expressed genes were identified from two independent transcriptomic datasets derived from cellular models of GD (Threshold:$\:\left|{\mathrm{l}\mathrm{o}\mathrm{g}}_{2}\left(\frac{geneExpressio{n}_{GD}}{geneExpresio{n}_{Control}}\right)\right|>1,p<0.05$, S Fig. 3). Of these, 196 genes were classified as metabolic genes, and 90 could be mapped onto reactions within the macrophage models. Enrichment analysis indicated that these differentially expressed metabolic genes were involved in pathways ranging from central carbon metabolism to broader lipid metabolic processes (Fig. 2A). Of the mapped genes, 61 were upregulated and 29 were downregulated. In total, 239 reactions were associated with the mapped genes, and their bounds were adjusted, representing the enzyme capacity. The resulting models were termed GD models, in contrast to the control models, which represent the healthy state.

Flux distributions for both GD and control models were then generated using EntropicFBA and visualised with t-SNE analysis (Fig. 2B). Two well separated clusters, each containing about 31 models, were observed corresponding to the GD and control groups. These clustered models were selected to represent the disease and healthy states in subsequent analyses, as they consistently reflected the gene expression patterns observed in the transcriptomic datasets.

#### Broad metabolic reprogramming in GD models

Differential flux analysis between GD and control models is presented in Fig. 2C. Among 4,326 reactions analysed, approximately 650 reactions were predicted to exhibit significant differences ($\:{\mathrm{l}\mathrm{o}\mathrm{g}}_{2}\left(\frac{{v}_{GD}}{{v}_{control}}\right)>$1, $\:p<0.05$) in flux levels. Of these, 50.2% of reactions showed increased fluxes, while the remaining 49.8% exhibited reduced fluxes in GD models relative to controls. Pathway enrichment analysis (Fig. 2D), based on subsystem classifications in Recon3D [41], revealed that these differentially active reactions spanned a wide range of metabolic pathways.

Notably, in GD models, central energy metabolism displayed a coordinated shift, with higher flux through glycolysis and gluconeogenesis but lower activity in the citric acid cycle, oxidative phosphorylation, and the pentose phosphate pathway. Lipid associated metabolism showed some of the most prominent changes. Increased flux was seen in sphingolipid and cholesterol metabolism, eicosanoid and steroid biosynthesis, bile acid synthesis and glutathione metabolism. In contrast, other lipid pathways were suppressed, including glycerophospholipid metabolism, fatty acid oxidation and fatty acid synthesis, together with reduced nucleotide interconversion and lower activity in related supporting pathways. Amino acid metabolism showed a mixed pattern, with increased flux in the pathways for tyrosine, tryptophan, alanine and aspartate, and reduced flux in the pathways for glycine, serine, threonine, methionine, cysteine, glutamate, arginine, proline, and the branched chain amino acids. This flux modelling results showed strong correspondence with the transcriptomic enrichment patterns (Fig. 2A), reflecting both the metabolic effects of dysregulated genes and additional pathway alterations revealed through the network structure of the model.

The flux modelling results showed strong correspondence with the transcriptomic enrichment patterns. Several pathways were identified by both approaches, including those related to carbohydrate metabolism, aerobic and mitochondrial respiration, fatty acid metabolism, eicosanoid and prostaglandin synthesis and vitamin associated processes. The overlap between these datasets indicates that the transcriptional alterations detected in Gaucher disease are reflected at the level of predicted metabolic flux. Additional pathways detected only by the modelling, particularly those involving broader lipid handling and cofactor usage, likely represent downstream consequences of the transcriptomic perturbation that become evident when the full metabolic network structure is considered.

### Metabolic alterations in GD models

#### Reprogrammed energy metabolism in GD models

**Fig. 4:**
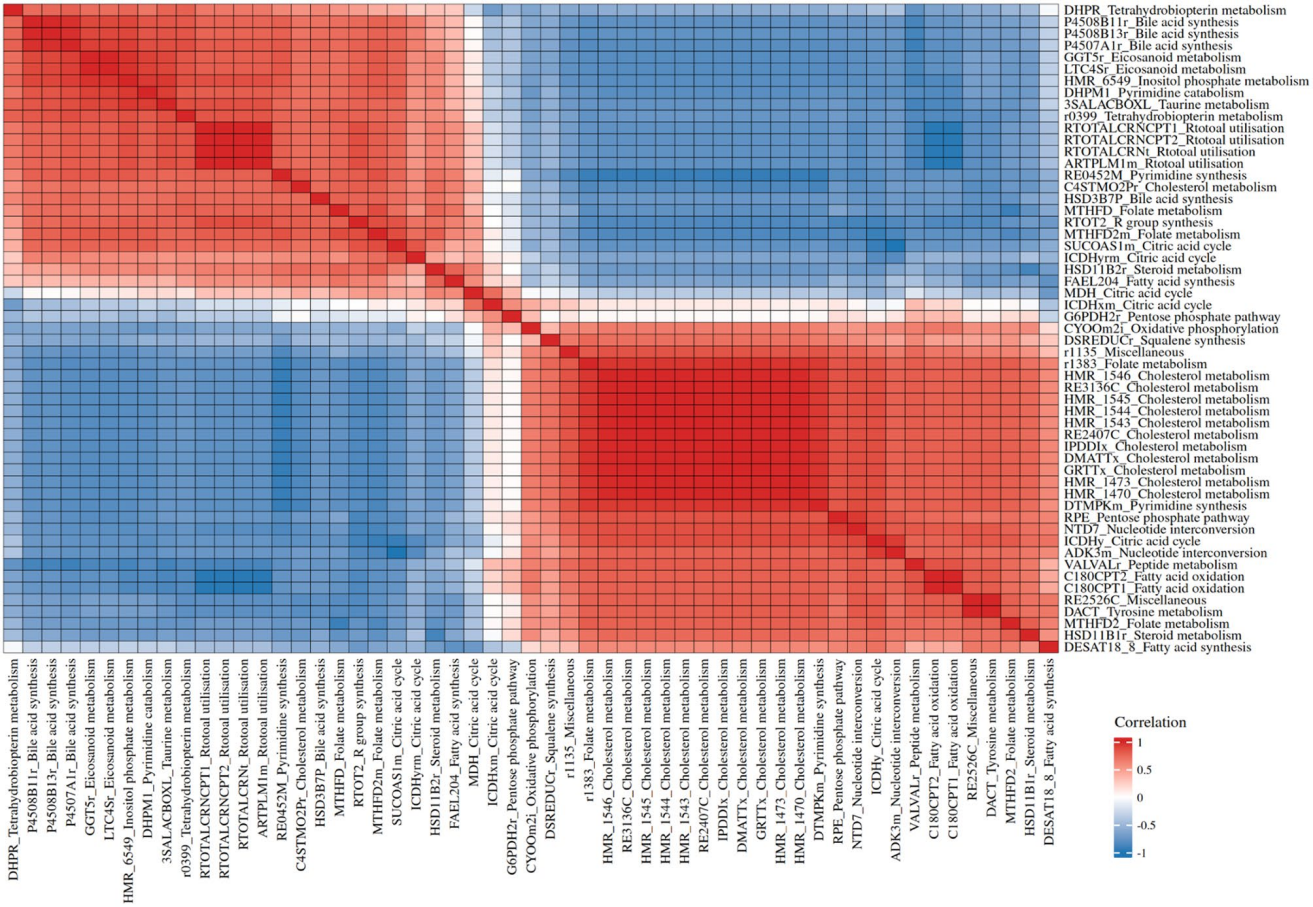
Disease-specific interaction in GD models. Clustered heatmap visualising pairwise Spearman correlations with GD-specific flux patterns. The top 55 reactions with the highest overall correlation to other reactions in the model are shown. Each cell represents the correlation coefficient between two reactions in GD models, with positive correlations shown in warmer colours and negative correlations in cooler colours. Reactions are annotated by their VMH IDs [66] and associated subsystems. Clustering reveals distinct modules of co-regulated metabolic processes, potentially reflecting coordinated functional responses in Gaucher disease

Results from differential flux analyses indicate a significant metabolic shift in energy metabolism after GBA1 deficiency, prompting further investigation into energy metabolism-related pathways. Robustness analysis using entropicFBA was performed to explore ATP contribution patterns in the models under varying energy demands (Fig. 3A). Across a wide range of ATP demand rates, the predicted proportional contribution of oxidative phosphorylation was consistently lower in the GD models compared with controls. This reduction was accompanied by a decrease in the contribution from the citric acid cycle, indicating a reduced reliance on mitochondrial ATP generating pathways. In contrast, glycolysis accounted for a larger share of total ATP production in the GD models. Mechanistically, this was linked to compromised pyruvate dehydrogenase activity, reduced mitochondrial ATP transport efficiency, and diminished activities of respiratory complexes I, II, and IV (S Fig. 4). In contrast, glycolytic enzymes such as pyruvate kinase and phosphoglycerate kinase were upregulated (S Fig. 4), underscoring a metabolic pivot to glycolysis under energy stress. These findings highlight the GD models’ reliance on glycolysis as a compensatory mechanism for oxidative phosphorylation insufficiency.

#### Defective sphingolipid metabolism in GD models

**Fig. 5:**
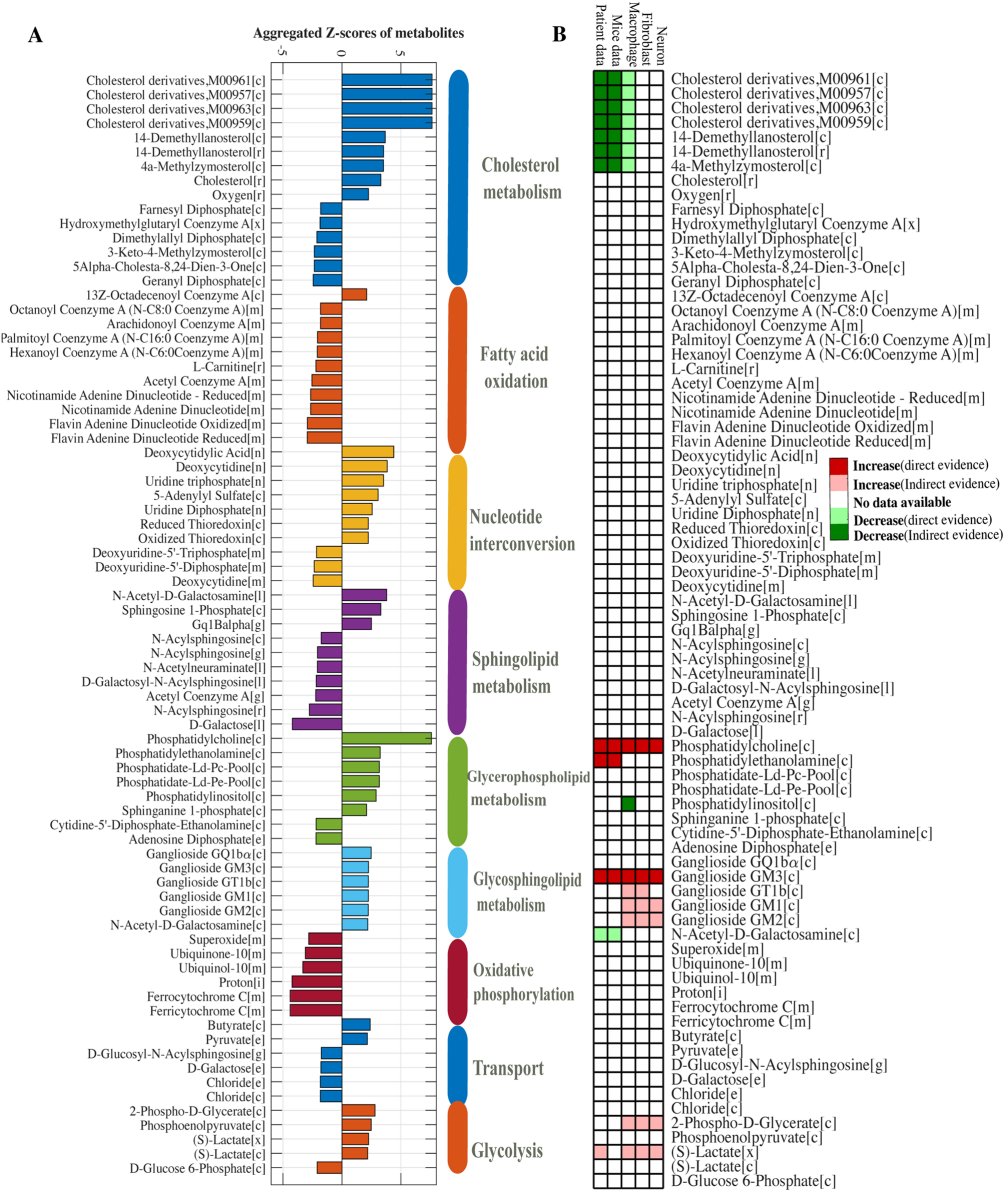
The identified reporter metabolites and literature validation. (**A**) Reporter metabolites significantly associated with transcriptional changes in GD models, grouped by metabolic subsystem. Metabolites were identified using the reporter metabolite algorithm [63], with bars indicating the mean log₂ fold change of gene expression between GD and control conditions. Subsystems are colour-coded to reflect their functional classification. (**B**) Literature- and data-driven evidence supporting the altered abundance of each reporter metabolite. Green and red cells represent direct evidence of increased or decreased levels, respectively, while light green and pink cells indicate indirect evidence. White cells denote no available data. This mapping integrates transcriptomic, metabolomic, and curated pathway knowledge to contextualise the predicted metabolic shifts

Differential flux analysis revealed significant alterations in sphingolipid metabolism in GD models. To explore this further, flux distributions of key enzymes involved in sphingolipid degradation, ceramide synthesis, and ganglioside biosynthesis were examined through robustness analysis (Fig. 3B). As expected, glucocerebrosidase activity was markedly reduced. Hexosaminidase flux was also decreased, while sphingomyelinase flux was significantly increased, suggesting compensatory degradation via alternative pathways. Enzymes involved in ceramide synthesis (serine palmitoyltransferase, sphinganine reductase, dihydroceramide synthase, dihydroceramide desaturase) showed uniformly elevated fluxes, indicating enhanced *de novo* synthesis of ceramide. Additionally, early steps in ganglioside synthesis (from ceramide to simple gangliosides like GM3 and GD3) show reduced flux in GD models, while downstream enzymes involved in complex ganglioside synthesis exhibit increased flux, suggesting a dysregulated or imbalanced ganglioside biosynthetic pathway in the context of *GBA1* deficiency. The qualitative pathway-level metabolic changes remained consistent under both lighter and stricter reaction constraints indicated by. differentially expressed genes (S Figs. 5 and 6), demonstrating that the overall conclusions are robust to variation in the chosen scaling factors for GD model generation.

#### Correlation analysis uncovers GD-specific metabolic interactions

Spearman correlation analysis was applied to reactions with differential fluxes between GD and control models to assess co-regulation patterns. This identified a subset of reactions with GD-specific correlation profiles (Fig. 4). This analysis revealed two major clusters with strong internal coherence. One cluster contained pathways related to cholesterol metabolism, steroid and bile acid synthesis, nucleoside interconversion and several vitamin-associated routes, all showing strong positive correlations with one another. The second cluster included pathways such as the citric acid cycle, oxidative phosphorylation, fatty acid oxidation and tetrahydrobiopterin metabolism, which also displayed tight internal positive correlations. These two clusters were strongly anti-correlated, indicating that increases in flux within cholesterol- and lipid-associated pathways were consistently accompanied by decreases in mitochondrial and central carbon metabolism pathways. Additional smaller groups, including amino acid and retinol utilisation pathways, showed intermediate patterns but followed the same broad structure of opposing metabolic modules. In combination with the transcriptomic and flux modelling analyses (Fig. 2), these correlations indicate that some metabolic alterations align with gene-level dysregulation, whereas others arise from the propagation of perturbations through the metabolic network.

### Network-based predictions of gaucher disease metabolism

#### Reporter metabolites of GD models

The reporter metabolites, defined as metabolites surrounded by reactions catalysed by significantly dysregulated genes, were predicted using the reporter metabolites algorithm [63]. The identified reporter metabolites were grouped into their respective metabolic subsystems, revealing prominent dysregulation in GD models compared to controls. The most affected subsystems were cholesterol metabolism, fatty acid oxidation, glycerophospholipid metabolism, glycosphingolipid metabolism, and oxidative phosphorylation (Fig. 5A).

Cholesterol metabolism was predicted to have increased synthetic activity, indicating a possible build-up of cholesterol intermediates within GD model. Consistent with the correlation analysis, this pattern was opposed by changes in oxidative phosphorylation. fatty acid oxidation showed a general decrease, especially for short and medium chain fatty acids. These results align with the flux-based analyses, providing internal cross validation of the predicted metabolic changes. To support this further, we compiled a literature-based evidence matrix across multiple biological sample types. In the corresponding heatmap, each metabolite is annotated with direct or indirect evidence from literature for increased or decreased abundance (Fig. 5B, S File 3). This analysis confirmed several computational predictions, particularly for phospholipids, acyl CoA intermediates and complex gangliosides, and also highlighted metabolites with limited prior evidence.

Interestingly, while the model predicted increased activity in cholesterol biosynthesis, clinical studies frequently report reduced plasma levels of total cholesterol, high-density lipoprotein cholesterol (HDL-C) and low-density lipoprotein cholesterol (LDL-C), in GD patients [67–70]. This apparent discrepancy reflects increased intracellular cholesterol synthesis coupled with impaired trafficking or export. These findings highlight the importance of distinguishing between intracellular metabolic activity and systemic metabolite levels. Collectively, these results provide mechanistic insight into dysregulated pathways and suggest that several reporter metabolites may represent promising biomarkers in GD.

#### Model-predicted modifier genes

The rMTA algorithm [64, 65] was used to identify genetic targets capable of transforming the metabolic states. When the objective was set to transform the disease state into the healthy state, rMTA failed to identify any gene knockouts capable of achieving this transition. In contrast, reversing the objective and transforming the healthy state into the disease state produced a set of gene knockouts that promoted a disease-like metabolic phenotype. These genes likely contribute to the metabolic alterations observed in GD and may act as modifiers that help explain gene–phenotype variability. The top gene knockouts with the highest transformation scores were extracted from the analysis and filtered them based on known associations with GD (Table 1).

Notably, these genes were enriched in pathways related to steroid and lipid metabolism (e.g., *ASAH1*, *AKR1C3*,* FABP1*,* GALE*,* CPT1A*,* AACS*), energy metabolism (*SUCLG1*,* SUCLG2*), and membrane or intracellular transport (*SLC5A1*). Several of these, such as *ASAH1* and *CPT1A*, are directly involved in lipid catabolism and mitochondrial fatty acid oxidation, which aligns with known metabolic disturbances in GD.

**Table 1:** Model-predicted modifier genes potentially aggravating metabolic perturbations in GD models

Gene symbol	Gene name	Related pathway
SLC5A1	Solute carrier family 5 member 1	Membrane or intracellular transport
ASAH1	N-acylsphingosine amidohydrolase 1	Steroid/Lipid metabolism
AKR1C3	Aldo-keto reductase family 1 member C3	Steroid/Lipid metabolism
SUCLG2	Succinate-coa ligase gdp-forming subunit β	Energy metabolism
FABP1	Fatty acid binding protein 1	Steroid/Lipid metabolism
SUCLG1	Succinate-coa ligase subunit α	Energy metabolism
GALE	UDP-galactose-4-epimerase	Steroid/Lipid metabolism
CPT1A	Carnitine palmitoyl transferase 1a	Steroid/Lipid metabolism
AACS	Acetoacetyl-coa synthetase	Steroid/Lipid metabolism

#### Transcription factor networks implicated by co-regulated metabolic modules

Spearman correlation analysis identified 50 co-regulated reaction sets unique to the GD models, 26 of which contained more than three reactions. The underlying cause of co-regulation among these reactions was assumed to stem from topological constraints within the metabolic network, or from genetic and regulatory information embedded in the model during its context-specific simulation. Several modules were linked to lipid metabolism, and transcription factor enrichment using ChEA3 showed that eight of the 26 modules were associated with dense transcriptional regulatory networks. One module contained *CYP* and *HSD* genes involved in bile acid and steroid biosynthesis and was regulated by an HNF4A-centred transcriptional network. Other modules connected cholesterol-related reactions with nucleotide metabolism, glutamate oxidation, and fatty acid transport and peroxisomal -oxidation. Across these modules, recurrent transcription factors such as HNF4A, MLXIPL, CREB3L3, and nuclear receptors (NR1I2, NR1I3, NR1H4) indicated shared transcriptional control of lipid and cholesterol regulatory programs in GD. Gene Ontology analysis of transcription factors from these networks showed significant enrichment of lipid-related biological processes, including intracellular receptor signalling, regulation of lipid metabolism and biosynthesis, and metabolic homeostasis (S File 3).

## Discussion

In this study, we applied a genome-scale modelling approach to simulate macrophage metabolism under GD and healthy conditions by integrating transcriptomic, proteomic, and bibliomic data. By comparing GD models with control models, we predicted significant metabolic reprogramming in GD, including shifts in energy metabolism and lipid metabolism, with cholesterol metabolism being consistently highlighted.

### Energy metabolism

Our model-based predictions indicate a pronounced shift in energy metabolism in GD models under increasing energy demand, characterised by diminished reliance on oxidative phosphorylation and a compensatory upregulation of glycolysis. Mechanistically, this ‘Warburg-like’ effect is attributed to impaired pyruvate dehydrogenase activity, reduced mitochondrial ATP transport, and dysfunction in respiratory complexes, all of which constrain mitochondrial ATP output.

Experimental studies in GD cellular models support the plausibility of these in silico predictions. Glucocerebrosidase deficiency disrupts lysosomal acidification and autophagic flux, impairing the clearance of damaged mitochondria and promoting mitochondrial stress [71]. Gaucher cells show elevated reactive oxygen species and reduced mitochondrial membrane potential [72], consistent with the model-predicted reduction in the activities of respiratory complexes I, II and IV and impaired ATP transport. Notably, a marked decrease in ATP synthesis linked to both complex I- and complex II/III-mediated phosphorylation has been observed despite normal activity of individual respiratory chain proteins [61], indicating that mitochondrial dysfunction reflects systemic bioenergetic collapse rather than isolated enzymatic defects. Elevated medium-chain acylcarnitine and oxidative stress markers in patient plasma further indicate impaired mitochondrial β-oxidation [15]. Coenzyme Q deficiency exacerbates electron-transport inefficiency and oxidative stress [72], while supplementation with Coenzyme Q or pharmacological chaperones partially restores oxidative phosphorylation. These findings align with the model, which identifies mitochondrial ATP transport and respiratory-complex function as key points of vulnerability.

Our simulations also predict that GD models progressively increase their dependence on glycolysis, reflected by higher flux through pyruvate kinase and phosphoglycerate kinase. This aligns with experimental observations of elevated glycolytic enzyme expression, increased lactate production, and enhanced glucose uptake in GD cells [15, 73, 74]. Increased basal glucose turnover has been reported clinically without corresponding rises in plasma glucose [18, 75], and resting energy expenditure is elevated by 24% − 44% in patients [14, 17, 18, 75]. Although enzyme replacement therapy can help decrease resting energy expenditure and improve nutritional status [75], it has also been associated with insulin resistance and weight gain [19, 20], pointing to persistent dysregulation of energy metabolism.

The predicted energy shift is also consistent with the macrophage activation state characteristic of Gaucher disease. Although Gaucher macrophages are classically described as M2-like based on surface markers (e.g., CD163 [76, 77]) and cytokines ( e.g., CCL18 [78]), accumulating evidence indicates that they simultaneously display metabolic and inflammatory features more typical of M1 activation. GD macrophages exhibit an inflammatory M1-like phenotype with increased cytokine secretion [79], oxidative stress [72] and a strong dependence on glycolysis [3]. Mitochondrial dysfunction promotes the “Warburg-like” metabolic program by limiting oxidative phosphorylation and enhancing redox signalling, while inflammatory cues further reinforce glycolytic reprogramming. Thus, the model-predicted suppression of mitochondrial ATP production and increased glycolysis aligns with, and may contribute to, the sustained inflammatory activation observed in GD macrophages.

In summary, glucocerebrosidase deficiency initiates a pathological cascade beginning with lysosomal dysfunction, followed by impaired mitochondrial quality control, oxidative phosphorylation inefficiency, and culminating in a glycolytic shift. This mechanistic insight may partly explain the observed link between GD and Parkinson’s disease, as both GD patients and carriers exhibit increased susceptibility to Parkinson’s disease [80–82]. In Parkinson’s disease, mitochondrial dysfunction is a key driver of dopaminergic neuron degeneration in the substantia nigra, with *GBA1*-associated Parkinson’s disease displaying more severe mitochondrial impairment than sporadic cases [83–85].

### Lipid metabolism

Model predictions indicate broader alterations across multiple lipid pathways. These alterations extend beyond sphingolipid metabolism into cholesterol and other lipid associated processes, forming a coherent pattern of lipid reprogramming in the GD models. These changes are best understood as secondary and adaptive responses that follow the disruption of sphingolipid metabolism caused by GBA1 deficiency. In this framework, the wider lipid landscape is remodelled not because each pathway is directly affected by the genetic defect, but because the primary disturbance in lysosomal lipid handling propagates through interconnected metabolic networks.

### Sphingolipid metabolism

Our model predicted fundamental alterations in sphingolipid metabolism following in silico inhibition of *GBA1* gene, consistent with reported lipidomic and cellular findings in GD. As expected, glucocerebrosidase flux was markedly reduced, reflecting the primary enzymatic defect and explaining the accumulation of glucosylceramide observed in patient plasma and experimental GD models [2, 28, 86, 87]. The predicted increased flux through sphingomyelinase suggests activation of alternative catabolic pathways to compensate for impaired glucosylceramide degradation, echoing experimental findings of redistributed glucosylceramide and secondary lipid accumulations outside lysosomes [2, 87–89]. The models also predict enhanced *de novo* ceramide synthesis, consistent with elevated levels of ceramide, dihexosylceramide, trihexosylceramide and phosphatidylglycerol reported in fibroblasts, macrophages and patient plasma [87, 89–92]. Reduced flux through the early steps of ganglioside biosynthesis (such as GM3 and GD3) contrasts with elevated GM3 levels found in fibroblasts [86, 90, 92, 93], pointing to possible cell-type differences or post-synthetic accumulation. In contrast, increased flux through later ganglioside synthesis steps in our models may reflect compensatory processing of accumulating intermediates or dysregulated pathway control. This aligns with experimental observations of GM1 and GM2 accumulation in fibroblasts and brain tissue from neuropathic GD cases [91, 92]. Experimental findings and our in silico predictions and highlight extensive remodelling of sphingolipid metabolism driven by GBA1 deficiency. They emphasise that sphingolipid dysregulation in GD extends beyond glucosylceramide accumulation to include broader alterations in ceramide production, sphingomyelin degradation and ganglioside biosynthesis. Collectively, these results provide mechanistic insight into the lipidomic imbalances observed in GD and underscore the intricate remodelling of sphingolipid pathways driven by GBA1 deficiency.

### Cholesterol metabolism

Across the systems-level analyses, cholesterol metabolism emerged as a central axis of metabolic dysregulation in GD models. Flux correlation analysis showed that cholesterol metabolism is tightly linked to steroid synthesis and inversely associated with oxidative phosphorylation and fatty acid oxidation. Reporter metabolite analysis indicated that upregulated genes in Gaucher macrophages map to cholesterol biosynthesis intermediates, suggesting increased transcriptional activation of this pathway. The modifier gene analysis identified regulators of steroid biosynthesis, and the co-regulated modules implicated cholesterol handling enzymes under shared transcriptional control. All these modelling results point to disruption of cholesterol homeostasis at multiple levels, driven by the underlying lysosomal dysfunction. The consistent prediction of increased cholesterol biosynthesis highlights a potentially measurable and therapeutically relevant metabolic response that may help identify disease modifiers, stratify patient subtypes, or guide targeted interventions in GD.

Clinical studies consistently report reduced circulating levels of total cholesterol, LDL-C, HDL-C and apolipoproteins in patients with Gaucher disease [67–69, 94]. In contrast, our computational models predict increased intracellular cholesterol biosynthesis in GD macrophages. This discrepancy can be explained by lysosomal dysfunction: glucosylceramide accumulation disrupts cholesterol trafficking, depleting cytosolic cholesterol and triggering a compensatory increase in biosynthesis.

These results are consistent with growing evidence that *GBA1* dysfunction perturbs cholesterol trafficking and metabolism at multiple levels. Lysosomal cholesterol accumulation has been observed in *GBA1*-deficient cells as a result of impaired efflux, partly due to disrupted NPC2 (intracellular cholesterol transporter 2) function and altered lysosomal pH [95–97]. This lysosomal trapping of cholesterol interferes with membrane fluidity, autophagic clearance, and mitochondrial integrity. Cholesterol accumulation can also disrupt the interaction between glucocerebrosidase and its trafficking partner LIMP-2 (lysosomal integral membrane protein-2), further reducing enzyme delivery to the lysosome [98–100]. Increased intracellular cholesterol further contributes to loss of glucocerebrosidase function by promoting endoplasmic reticulum associated degradation of the mutant enzyme, adding an additional layer of trafficking impairment [101]. In neurons, these disruptions are further implicated in the formation of cholesterol-rich multilamellar bodies that act as nucleating sites for -synuclein aggregation, which is a hallmark of Lewy body pathology in *GBA1*-associated Parkinson’s disease [98, 102, 103]. Cholesterol-rich environments may also impair vesicular trafficking, autophagic flux, and lipid raft dynamics, contributing to synaptic dysfunction and neuroinflammation [98].

Additional biochemical evidence suggests that, in the context of lysosomal overload, cholesterol can be aberrantly processed into cholesteryl-D-glucoside by non-lysosomal glucocerebrosidase activity (*GBA2*), contributing to further lipid imbalance [88, 104]. Our computational model predicts activation of steroid biosynthesis under these conditions, consistent with alternative cholesterol-handling pathways becoming engaged when lysosomal function is compromised. The model also predicts increased activity of reactions catalysed by *CYP* and *HSD* family genes, mirroring transcriptional responses observed in metabolic overload states such as obesity [105, 106]. These similarities suggest that lipid accumulation in Gaucher macrophages may activate shared lipid-sensing pathways. These responses are likely orchestrated by sterol-sensitive transcription factors, particularly members of the SREBP and LXR families, which regulate cholesterol biosynthesis, efflux and broader lipid balance [107, 108]. Similar regulatory patterns have been reported in other lysosomal storage disorders, including Niemann Pick type C, where sphingolipid accumulation activates cholesterol biosynthesis through SREBP signalling [109, 110].

The predicted increase in intracellular cholesterol biosynthesis raises an important question, given the consistently reduced circulating cholesterol in GD. The explanation lies in the distinction between systemic lipid levels and intracellular sterol availability. In *GBA1* deficiency, cholesterol becomes sequestered within lysosomes and is therefore unavailable in the cytosol and endoplasmic reticulum. This depletion activates cholesterol and steroid biosynthesis pathways to maintain essential cellular functions.

These results suggest that cholesterol dysregulation is a central feature of Gaucher macrophage biology, and the model-predicted increase in sterol biosynthesis constitutes a testable hypothesis. Several experimental approaches could be used to validate both the predicted metabolic changes and the proposed mechanisms. The predicted increase in cholesterol and steroid biosynthesis could be assessed using lipidomics and stable isotope tracing to quantify de novo sterol synthesis [111], while changes in pathway capacity could be evaluated through siRNA knockdown or proteomic analysis of *CYP* and *HSD* enzymes [112]. Mitochondrial respiration assays would help determine whether alterations in energy metabolism occur alongside the predicted lipid rewiring [71]. Targeted experiments could also examine the mechanistic explanation suggested by our model. Subcellular lipidomics, filipin imaging and perturbation of NPC2 or related trafficking pathways could test whether cholesterol becomes sequestered in lysosomes and whether restoring efflux normalises biosynthetic activity [113]. These approaches provide focused strategies for evaluating the metabolic predictions generated by the model and the biological rationale underlying them.

### Putative modifiers and transcription factors

Application of the rMTA algorithm predicted *SLC5A1*, *ASAH1*, *AKR1C3*, *SUCLG2*, *FABP1*, *SUCLG1*, *GALE*, *CPT1A*, and *AACS* as top candidate genes whose knockdown would exacerbate metabolic differences between GD and control models. These genes converge on pathways related to lipid metabolism, central carbon metabolism, and energy homeostasis, highlighting potential metabolic nodes that may modify disease severity.

Notably, *ASAH1*, which encodes acid ceramidase, metabolises ceramide into sphingosine and contributes to the formation of glucosylsphingosine, a cytotoxic lipid elevated in all GD forms [2, 114–117]. Although *ASAH1* gene inhibition has been proposed as a means to reduce glucosylsphingosine, our model suggests that diminishing *ASAH1* activity may worsen global metabolic imbalance. Experimental studies support this caution: *ASAH1* deficiency increases ceramide and cholesterol accumulation, disrupts lipid homeostasis and compromises neuronal viability [118, 119]. These complementary findings suggest that *ASAH1* may act as a metabolic stabiliser rather than a simple therapeutic target, and that its modulation requires careful evaluation. *CPT1A*, another high-scoring rMTA gene, is a rate-limiting enzyme for mitochondrial fatty acid import and oxidation. Its identification is notable given the energetic and mitochondrial impairments observed in GD [120]. Reduced *CPT1A* activity could aggravate mitochondrial dysfunction by limiting fatty acid utilisation and increasing reliance on glycolysis, a shift already predicted by our models and supported by experimental evidence [120]. Although *CPT1A* has not been directly implicated in GD, its central role in cellular energetics suggests potential relevance as a putative disease modifier, warranting targeted investigation. The remaining rMTA-identified genes are not directly linked to GD in current literature but are involved in metabolic processes relevant to lipid and energy regulation, warranting further study.

Beyond metabolic enzymes, our analysis also highlighted transcription factors that may coordinate the broader metabolic reprogramming observed in Gaucher macrophages. Regulators such as HNF4A, CREB3L3 control cholesterol, bile acid and lipid metabolism [121, 122]. SPI1, a key myeloid transcription factor, may contribute to macrophage-specific adaptations [123]. Their involvement in GD has not yet been demonstrated experimentally, but the predictions raise testable hypotheses regarding regulatory circuits integrating lipid and nutrient sensing.

These results provide mechanistically grounded and testable hypotheses for metabolic modifiers of GD. Their translational relevance lies in identifying metabolic nodes such as *ASAH1*, *CPT1A* and associated transcriptional regulators that may influence disease progression, therapeutic response or biomarker development, warranting targeted experimental investigation.

### Limitations and future perspective

The current GD metabolic models have limitations that constrain their quantitative accuracy. The models in this study were reconstructed from transcriptomic data of monocyte derived macrophages rather than from tissue resident macrophages or primary patient samples. Although these cells are convenient for experiments and represent the myeloid lineage most affected in GD, they do not fully reflect the environment or functional state of macrophages in human tissues. Monocyte derived macrophages develop in culture and therefore lack the local signals, such as oxygen level, cytokine balance and metabolic gradients, that shape tissue specific behaviour.

While qualitative nutrient uptake predictions align reasonably with experimental data, overall predictive power is modest, as reflected by moderate correlations with measured exchange rates. A key discrepancy arose in amino acid exchange predictions: essential amino acids (e.g., L-histidine, L-lysine) were modelled as strictly imported, yet appeared elevated in spent medium, suggesting net secretion. This may stem from undefined components in serum supplements, whose uncharacterised composition complicates interpretation. Limited metabolite coverage in the validation dataset (14 metabolites) further restricts evaluation accuracy, as unmeasured uptake could falsely imply secretion.

Additionally, the use of exometabolomic data from mouse RAW264.7 cells limits disease relevance. Lipid metabolism is also under-specified, with many reactions represented at the lipid class level rather than as specific molecular species, reducing biological resolution. To enhance the predictive accuracy of metabolic models for GD, it is essential to integrate more comprehensive experimental datasets. These should include intracellular metabolite concentrations, enzyme activity profiles, and metabolic flux measurements obtained from patient-derived macrophages. Incorporating such data will enable more precise constraint definition and improve simulation fidelity. Furthermore, expanding the representation of lipid metabolism to include well-characterised lipid species will facilitate prediction of specific molecular changes relevant to disease mechanisms. Future research should prioritise systematic data collection from GD-affected human cells and the continued refinement of model granularity to support both mechanistic understanding and therapeutic development.

## Conclusion

This work used genome scale metabolic modelling to map the metabolic alterations associated with GBA1 deficiency in macrophages. Integration of multi omics data revealed coordinated rewiring of energy and lipid metabolism, marked by reduced oxidative phosphorylation, increased glycolytic dependence and activation of cholesterol biosynthesis. These changes appear to arise from lysosomal dysfunction and may be regulated by lipid sensitive transcription factors including SREBP2, LXRs and HNF4A.

Although patients with GD show reduced circulating cholesterol, the models predict intracellular sterol depletion and compensatory biosynthesis, pointing to a separation between systemic lipid profiles and cellular metabolic needs. The identification of *ASAH1* and *CPT1A* as candidate disease modifiers highlights metabolic nodes that could influence disease progression or therapeutic response. Overall, the modelling framework generates mechanistically grounded predictions and provides a basis for developing biomarkers and intervention strategies that can be evaluated experimentally.

## Supplementary Information

Below is the link to the electronic supplementary material.

Supplementary Material 1

Supplementary Material 2

Supplementary Material 3

## Data Availability

All data generated or analysed during this study are included in this published article and its supplementary information files. All the code generating the results in this article is available at: https://github.com/opencobra/COBRA.papers.

## Footnotes

1 Rarely, a neonatal collodion baby phenotype has been reported, characterised by severe skin-barrier defects and early lethality, though this is not a recognised subtype [5]. Additionally, a clinical manifestation resembling type III GD is observed during deficiency of Saposin C, the lysosomal activator protein assisting GBA1 in glucosylceramide hydrolysis [6].

2 CLN8 gene encodes a transmembrane protein localised to the endoplasmic reticulum (ER) and ER-Golgi intermediate compartment [9]. The GRIN2B gene encodes the NMDA-type glutamate receptor subunit 2B [10]. SCARB2 gene encodes LIMP2, which is involved in G trafficking [7]. PSAP gene encodes prosaposin, the precursor of Saposin C [7]. UGCG encodes glucosylceramide synthase [8]. PGRN gene encodes progranulin, which regulates lysosomal function and inflammation [11, 12, 13].

## Abbreviations

DMEM: Dulbecco’s Modified Eagle Medium
entropicFBA: Entropic Flux Balance Analysis
FBA: Flux Balance Analysis
FPKM: Fragments Per Kilobase of transcript per Million mapped reads
GD: Gaucher Disease
LC-MS: Liquid Chromatography-Mass Spectrometry
MS: Mass Spectrometry
PC: Phosphatidylcholine
PD: Parkinson’s disease
PE: Phosphatidylethanolamine
PS: phosphatidylserine
rMTA: robust Metabolic Transformation Algorithm
VMH: Virtual Metabolic Human database
