# Supplementary material for: Constraint-based modelling of metabolic dysregulation in Gaucher disease: mitochondrial dysfunction and disrupted cholesterol homeostasis
Source: Orphanet J Rare Dis. 2026 Jan 20;21:102. doi: 10.1186/s13023-026-04206-8 (PMC13005512; doi:10.1186/s13023-026-04206-8)
Supplement: Supplementary file 1 — Supplementary Material 1 [file 13023_2026_4206_MOESM1_ESM.docx]

Table of Contents

[Supplementary Material Overview 2](#_Toc215052241)

[Detailed mathematical explanation for FBA and entropicFBA 3](#_Toc215052242)

[Detailed mathematical explanation for rMTA algorithem 5](#_Toc215052243)

[Detailed mathematical explanation for reporter metabolite analysis 6](#_Toc215052244)

[S Table 1 User-defined parameters in 'XomicsToModel' pipeline 7](#_Toc215052245)

[S Table 2 Biochemical consistency check methods 7](#_Toc215052246)

[S Figure 1 Exometabolomic data driven selection of macrophage models. 8](#_Toc215052247)

[S Figure 2 Model prediction versus experimental measurement on metabolite exchange 9](#_Toc215052248)

[S Figure 3 Differentially expressed metabolic genes in Gaucher datasets (threshold log2FC > 1 or log2FC < -1) 9](#_Toc215052249)

[S Figure 4 Robustness Analysis over critical enzymes in energy metabolism under varying ATP demand. 10](#_Toc215052250)

[S Figure 5 Impact of Alternative DEG Scaling Factors on Model Predictions (lighter constraints) 11](#_Toc215052251)

[S Figure 6 Impact of Alternative DEG Scaling Factors on Model Predictions (stricter constraints) 14](#_Toc215052252)

# Supplementary Material Overview

#### The supplementary materials accompanying this manuscript are organised into three files:

#### **Supplementary File 1** provides all input layers required to reproduce the macrophage metabolic model with the XomicsToModel pipeline. This Excel workbook includes active genes, active reactions, present metabolites, reaction updates and additions, reaction constraints, essential amino acids, coupled reactions, and the corresponding cell culture conditions.

#### **Supplementary File 2** contains model-derived datasets and outputs that are too large to present in a document format. These include: (S1) the exometabolomic dataset used to optimise model predictive performance; (S2) detailed predictive accuracy results; (S3) model-predicted co-regulated reaction sets; (S4) reporter metabolites; and (S5) reporter metabolites accompanied by curated literature evidence.

#### **Supplementary File 3** provides additional figures and smaller tables that support the main text, including benchmarking metrics, flux prediction comparisons, differential expression analyses, robustness analyses, and predicted exchange profiles, as shown throughout the provided PDF

# Detailed mathematical explanation for FBA and entropicFBA

#### **Flux balance analysis** FBA estimates steady-state metabolic fluxes by solving a linear optimisation problem:

$$\begin{matrix} max & c^{T}v & \\ s.t. & S.v & =0, (1) \\ l & \leq vi\leq& u. (2) \end{matrix}$$

Where $S\in\mathbb{Z}^{m\times n}$ is stoichiometric matrix $v$ is the vector of reaction fluxes, $c$ is the objective coefficient vector, indicating the biological target that the network aim to achieve, $l$ and $u$ are the lower and upper bounds on each reaction rate. Equation (1) imposes the mass balance constraint under steady-state conditions, ensuring that, for each metabolite—including those involved in exchange reactions—the total rate of production plus input from the environment equals the total rate of consumption plus output to the environment. This reflects an assumption of constant metabolite concentrations over time, with no net accumulation or depletion. Inequality (2), the reaction bounds, imposes directional constraints on fluxes based on thermodynamic feasibility or quantitative constraints from experimental measurements. The objective to maximise $c^{T}v$ corresponds to maximising the sum of fluxes through one or more reactions.

#### **Entropic flux balance analysis** While FBA has been widely used, it suffers from key limitations. In cases where biological information is sparse or incomplete, FBA tends to produce biased flux distributions, often dominated by fluxes pinned at their upper or lower bounds. This can lead to unrealistic or overly rigid predictions that fail to capture the metabolic flexibility observed in living systems. Also, determining an appropriate objective function in FBA is challenging, given that the true metabolic goals of cells may vary across contexts and are not always well-defined. To address these limitations, we employed Entropic flux balance analysis (entropicFBA) [64, 67], which integrates entropy maximisation into the FBA framework to promote more biologically plausible flux distributions. The optimisation problem is represented by

$${min \atop v_{f}, v_{r},w}{g\circ v_{f}ᵀ\cdot log\left( v_{f} \right)+g\circ v_{r}ᵀ\cdot log\left( v_{r} \right)+cₑᵀ\cdot w+½\left( v-h \right)ᵀ\cdot H\cdot\left( v-h \right) \atop}$$

$s.t.$ N · ($v_{f}$- $v_{r}$) + B · $w$ = b, : $y_{N}$

C · ($v_{f}$- $v_{r}$) ≤ d, : $y_{C}$

l ≤ [$v_{f}$- - $v_{r}$; $w$] ≤ u, : $Z_{v}$

0 ≤ $v_{f}$, :$Z_{v_{f}}$

0 ≤ $v_{r}$, :$Z_{v_{r}}$

where the objective function combines three components. The terms $g\circ v_{f}^{T}\cdot log\left( v_{f} \right)$ and $g\circ v_{r}^{T}\cdot log\left( v_{r} \right)$ represent the entropy of unidirectional forward and reverse fluxes, respectively. Here, $g$ is a strictly positive weight (default: 2) applied to maximise the entropy of internal fluxes. denotes the entrywise (Hadamard) product of two vectors and $\cdot$ denotes the scalar product of two vectors. Since all reversible reactions can be represented as the difference between forward ($v_{f}$) and reverse ($v_{r}$) unidirectional fluxes, the net internal flux is defined as $v=v_{f}-v_{r}$. The term $c_{e}^{T}\cdot w$ accounts for the optimisation of external fluxes, where $c_{e}$ is a real-valued linear objective coefficient for external flux, and $w$ is the external flux vector. The quadratic penalty term $\frac{1}{2}\left( v-h \right)^{T}\cdot H\cdot\left( v-h \right)$ penalises deviations between predicted and experimentally measured fluxes, where $h$ is a reference flux vector (e.g., from the metabolomic data), and $H$ is a diagonal matrix that assigns penalty weights to deviations [64].

Several constraints are imposed. Mass balance is enforced through the equality $N\cdot\left( v_{f}-v_{r} \right)+B\cdot w=b$, ensuring that, at steady state, the net production and consumption of internal metabolites are balanced, and that for external metabolites, the sum of production and import equals the sum of consumption and export. Here, $N$ and $B$ are the stoichiometric matrices for internal and external reactions; $b$ representing the net production or consumption vector. Coupling constraints, expressed as $C\cdot\left( v_{f}-v_{r} \right)\leq d$, capture relationships among reaction fluxes, such as regulatory dependencies. Bound constraints, expressed as $l\leq\left[ v_{f}-v_{r};w \right]\leq u$, define lower and upper bounds on reaction fluxes. Non-negativity constraints are also enforced on the forward and reverse unidirectional fluxes: $0\leq v_{f}$ and $0\leq v_{r}$. Altogether, this defines a strictly convex optimisation problem, ensuring a unique and well-defined solution. Entropy maximisation produces the least biased prediction, given the available data.

# Detailed mathematical explanation for rMTA algorithem

Mathematically, rMTA solves a mixed-integer quadratic programming (MIQP) problem defined as:

$$min\left( 1-\alpha\right)\sum_{i\in R_{S}} \left( v_{i}^{\text{ref}}-v_{i} \right)^{2}+\frac{\alpha}{2}\sum_{i\in R_{F}} y_{i}+\frac{\alpha}{2}\sum_{i\in R_{B}} y_{i}$$

$$\begin{matrix} s.t. & S\cdot v=0, & & & \\ & v_{i}^{min}\leq v_{i}\leq v_{i}^{max}, & & & \\ & v_{i}-y_{i}^{F}\left( v_{i}^{\text{ref}}+\varepsilon_{i} \right)-y_{i}v_{i}^{min}\geq0, & & i\in R_{F,} & \\ & y_{i}^{F}+y_{i}=1, & & i\in R_{F,} & \\ & v_{i}-y_{i}^{B}\left( v_{i}^{\text{ref}}-\varepsilon_{i} \right)-y_{i}v_{i}^{max}\leq0, & & i\in R_{B,} & \\ & y_{i}^{B}+y_{i}=1, & & i\in R_{B.} & \end{matrix}$$

where, rMTA minimises a weighted sum of two objectives: the squared deviation between the perturbed and reference flux distributions over a selected subset of reactions, and the number of reactions permitted to deviate significantly from the reference. subject to steady-state mass balance $S\cdot v=0$, reaction flux bounds $v_{i}^{min}\leq v_{i}\leq v_{i}^{max}$, and a series of logical constraints that define deviation thresholds around the reference fluxes. In this formulation, $v^{ref}$represents the reference flux distribution (e.g., from control models via flux sampling), and $v$represent the flux distribution of the GD model. $y_{i}\in\left\{ 0,1 \right\}$indicates whether reaction $i$deviates beyond a tolerance $\epsilon_{i}$(default value: 0) and $\alpha\in\left[ 0,1 \right]$controls the trade-off between minimising flux deviation and minimising network rewiring ( a default value of $\alpha=0.66$was used in this study).

rMTA was applied across all GD models using the flux distribution from control models as references. For each gene, a robust transformation score (rTS) was computed by integrating three metrics: (i) the best-case transformation score (bTS), which quantifies how effectively the gene knockout moves the flux distribution towards the healthy state; (ii) the worst-case score (wTS), which assesses the risk of exacerbating the disease phenotype; and (iii) the MOMA-based transformation score (mTS), derived from the Minimisation of Metabolic Adjustment (MOMA) framework, which measures the total deviation from the unperturbed GD flux profile. These metrics were combined to prioritise gene deletions that robustly redirect the metabolic state towards a healthy configuration while minimising off-target effects.

# Detailed mathematical explanation for reporter metabolite analysis

This reporter metabolite analysis represents the metabolic system as a bipartite graph, linking enzyme-catalysed reactions to their associated metabolites. By mapping transcriptional changes onto this graph, the algorithm quantifies the aggregate differential expression of enzymes surrounding each metabolite within the metabolic model.

Each metabolite node in the network was assigned a score based on the normalised transcriptional response of its neighbouring enzymes. For a given metabolite $m$, let $G_{m}=g_{1},g_{2},...,g_{\kappa}$denote the set of $\kappa$genes encoding enzymes that catalyse reactions involving metabolite $m$. The aggregated Z-score for the metabolite was computed as:

$$Z_{metabolite}=\sum_{i=1}^{\kappa} Z_{g_{i}}$$

where $Z_{g_{i}}$represents the differential expression Z-score of gene $g_{i}$, calculated by comparing the gene’s expression change, $log\left( \frac{g_{i_{disease}}}{g_{i_{control}}} \right)$, to the distribution of changes across all genes.

To account for the background distribution of Z-scores in the network, the aggregated score was normalised by subtracting the mean $\mu_{\kappa}$and dividing by the standard deviation $\sigma_{\kappa}$, both calculated from multiple random gene sets of the same size $\kappa$:

$$Z_{metabolite}^{norm}=\frac{Z_{metabolite}-\mu_{\kappa}}{\sigma_{\kappa}}$$

This aggregated Z-score reflects the extent to which a metabolite is surrounded by enzymes that are collectively and significantly regulated, compared to random expectation. Metabolites with the highest aggregated Z-scores—reflecting coordinated transcriptional responses among neighbouring genes—are defined as reporter metabolites. These metabolites serve as indicators of metabolic regions under strong transcriptional regulation in response to perturbations. Statistical significance was assessed by comparing observed Z-scores to a background distribution generated through randomisation, ensuring robustness of the findings.

# S Table 1 User-defined parameters in 'XomicsToModel' pipeline


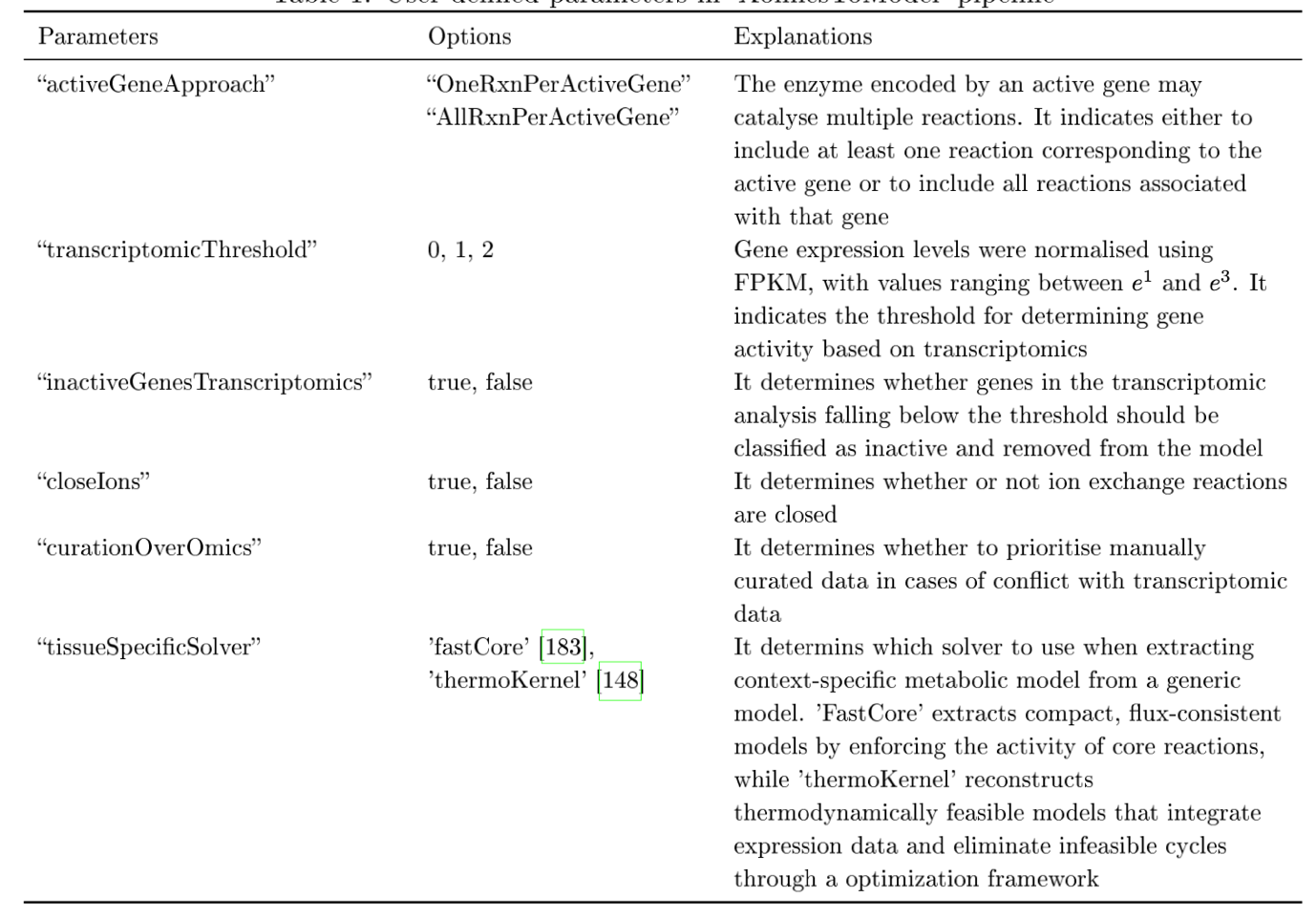


# S Table 2 Biochemical consistency check methods


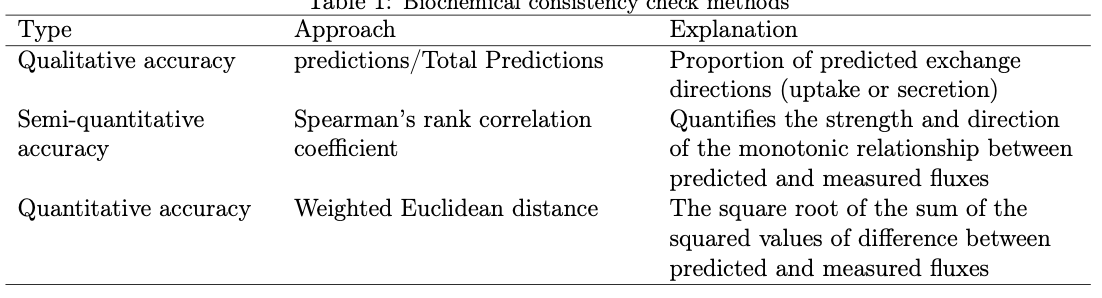


# S Figure 1 Exometabolomic data driven selection of macrophage models.

**
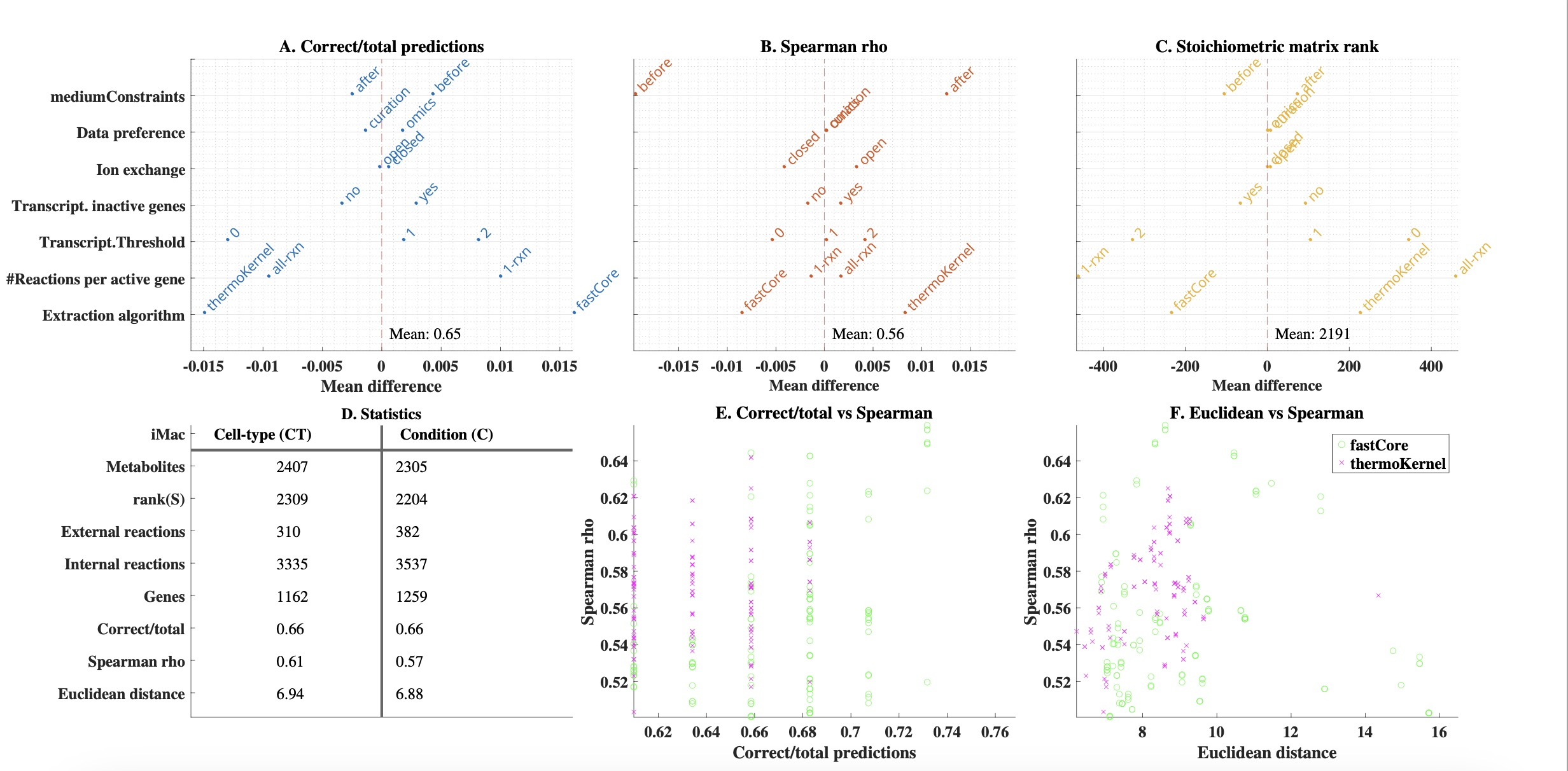
**

S Figure 1. Exometabolomic data driven selection of macrophage models. **(A-C)**: The effects on predictive accuracy and model size of changing ‘XomicsToModel’ pipeline parameters, averaged over all candidate objectives, excluding outliers. Specifically, **(A)** Qualitative accuracy of predictions, given by the number of correct predictions of secretion/uptake/neither divided by the number of total predictions. **(B)** Quantitative accuracy of predictions, given by the Euclidean norm of the differences between predicted and measured exchange fluxes, weighted by the inverse of the square of the measured exchange flux. **(C)** Average model size, given by the rank of the stoichiometric matrix. **(D)** Comparison between models prioritising manual curation over omics data (cell-type-specific models) and those prioritising omics data over manual curation (condition-specific models). **(E)** Qualitative and quantitative accuracy of exchange flux prediction can be more or less correlated. Given the same objective, there can be a large difference between the qualitative accuracy of the predictions, depending on the model. **(F)** Smaller models tend to give rise to higher qualitative predictive accuracy.

# S Figure 2 Model prediction versus experimental measurement on metabolite exchange

**
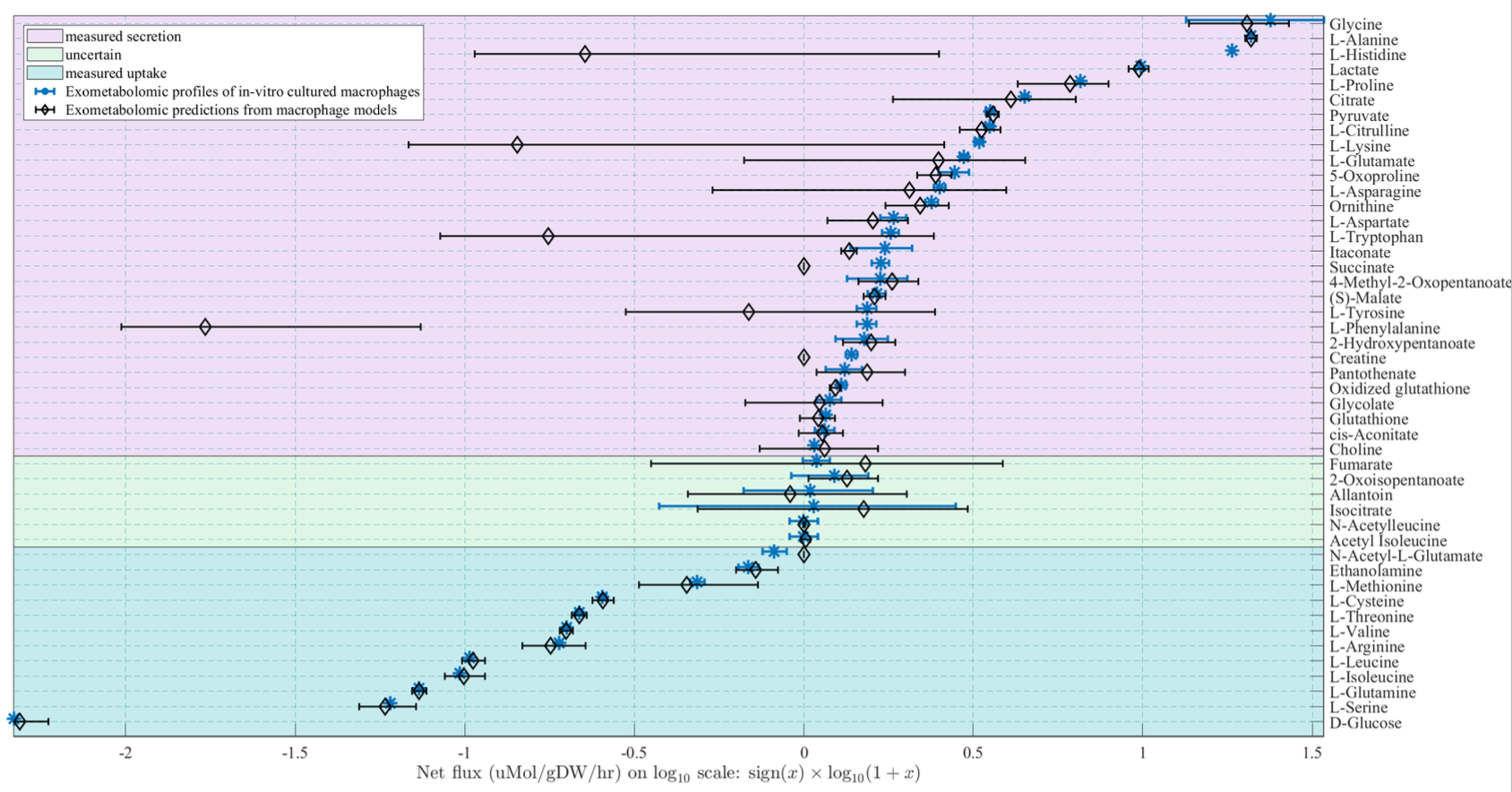
**

S Figure 2 Model prediction versus experimental measurement on metabolite exchange. Predicted fluxes are shown as black diamonds with error bars (mean ± standard deviation from 150 macrophage models). Experimental measurements are shown as blue asterisks with error bars representing one standard deviation; metabolites whose error bars intersect zero are considered not exchanged with the medium. Qualitative agreement is stronger for uptake predictions (given secretion constraints) than for secretion predictions (given uptake constraints).

# S Figure 3 Differentially expressed metabolic genes in Gaucher datasets (threshold log2FC > 1 or log2FC < -1)


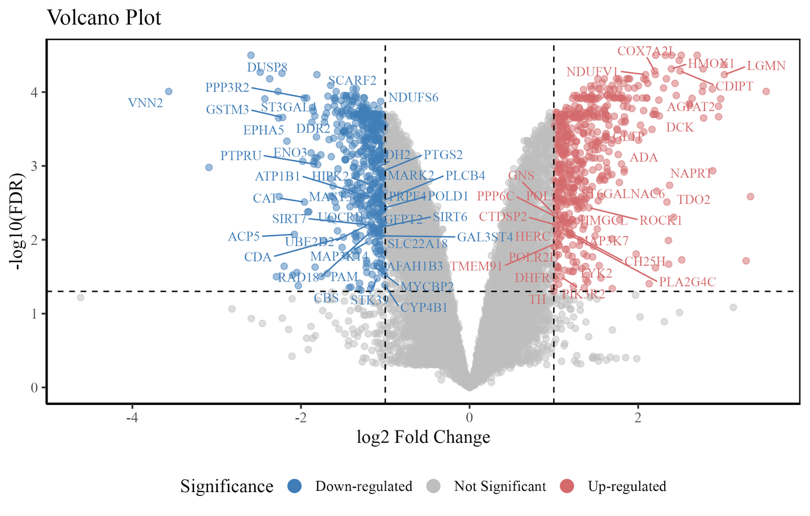


S Figure 3 Gene differential analysis from in-vitro models of Gaucher disease. Dataset: GSE183484 comprises gene expression data from macrophages differentiated from induced pluripotent stem cells (iPSCs) derived from a type 2 Gaucher disease patient and healthy controls. Dataset: GSE13675 originates from human bone marrow mesenchymal stromal cells with or without glucocerebrosidase inhibition.( threshold log2FC > 1 or log2FC < -1)

# S Figure 4 Robustness Analysis over critical enzymes in energy metabolism under varying ATP demand.


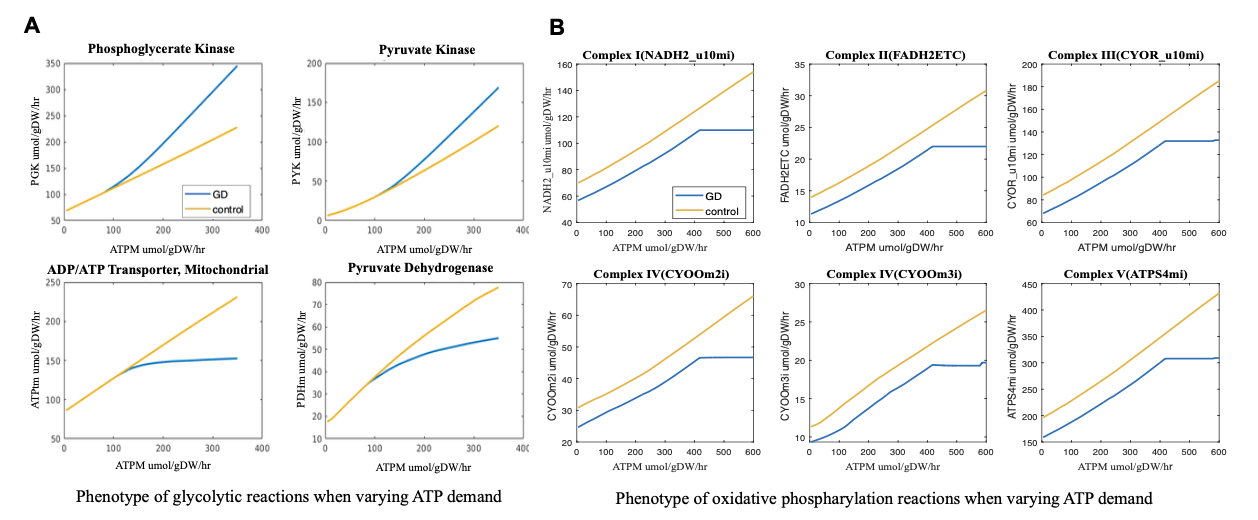


S Figure 4 Robustness Analysis over critical enzymes in energy metabolism under varying ATP demand. (A) Glycotic reactions including phosphoglycerate kinase (PGK), pyruvate kinase (PYK), mitochondrial ADP/ATP transporter (ATPtm), and mitochondrial pyruvate dehydrogenase (PDHm). (B) Oxidative phosphorylation reactions including mitochondrial Complex I (NADH2_u10mi), Complex II (FADH2ETC), Complex III (CYOR_u10mi), Complex IV (CYOOm2i and CYOOm3i(superoxide-generating)), and Complex V (ATPS4mi).

# S Figure 5 Impact of Alternative DEG Scaling Factors on Model Predictions (lighter constraints)


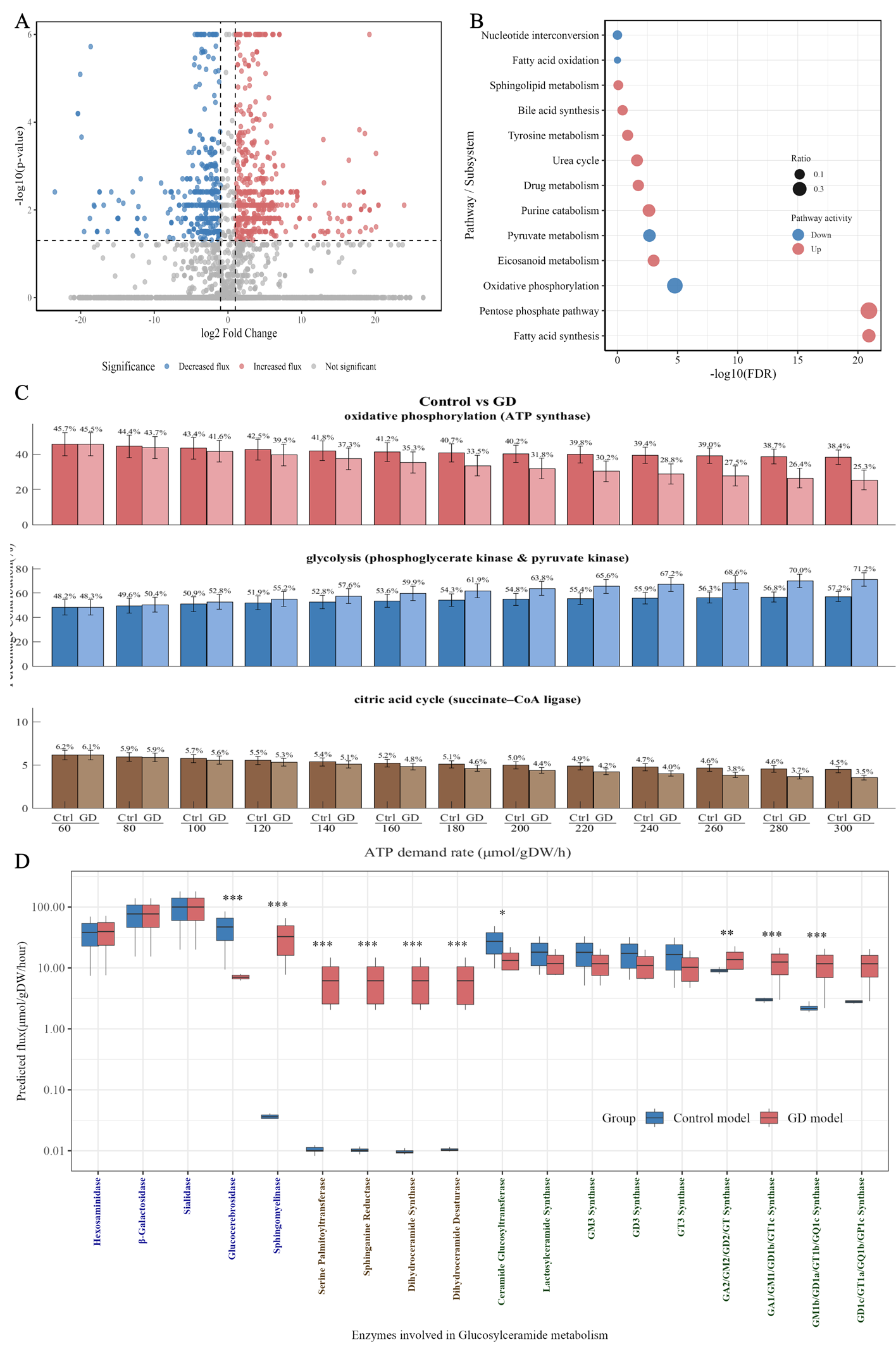


S Figure 5 Impact of Alternative DEG Scaling Factors on Model Predictions (lighter constraints). A lighter constraint was applied to generate GD models (70 % for down-regulated reactions and 130 % for up-regulated reactions). The qualitative metabolic changes highlighted in the main text, particularly at the pathway level, remained consistent across all tested parameter settings. (A) Volcano plot showing fold changes in reaction fluxes between GD and control models as predicted by entropicFBA. (B) Pathway enrichment analysis of reactions with significantly altered fluxes, mapped to metabolic subsystems defined in Recon3D. (C)Predicted ATP contribution from the three main energy-producing pathways. Relative contributions of glycolysis, the citric acid cycle and oxidative phosphorylation to total ATP production in control and GD models, estimated using entropicFBA. (D)Predicted activity of key enzymes in sphingolipid metabolism. Flux distributions for enzymes across the three major branches of sphingolipid metabolism in control and Gaucher disease models are shown on a log_10_ scale (umol/gDW/hour). X-tick labels are colour-coded to indicate pathway assignment: sphingolipid degradation (dark blue), de novo ceramide synthesis (brown) and complex ganglioside synthesis (dark green). Statistical significance is indicated as follows: p < 0.05 (*), p < 0.01 (**), and p < 0.001 (***).

# S Figure 6 Impact of Alternative DEG Scaling Factors on Model Predictions (stricter constraints)


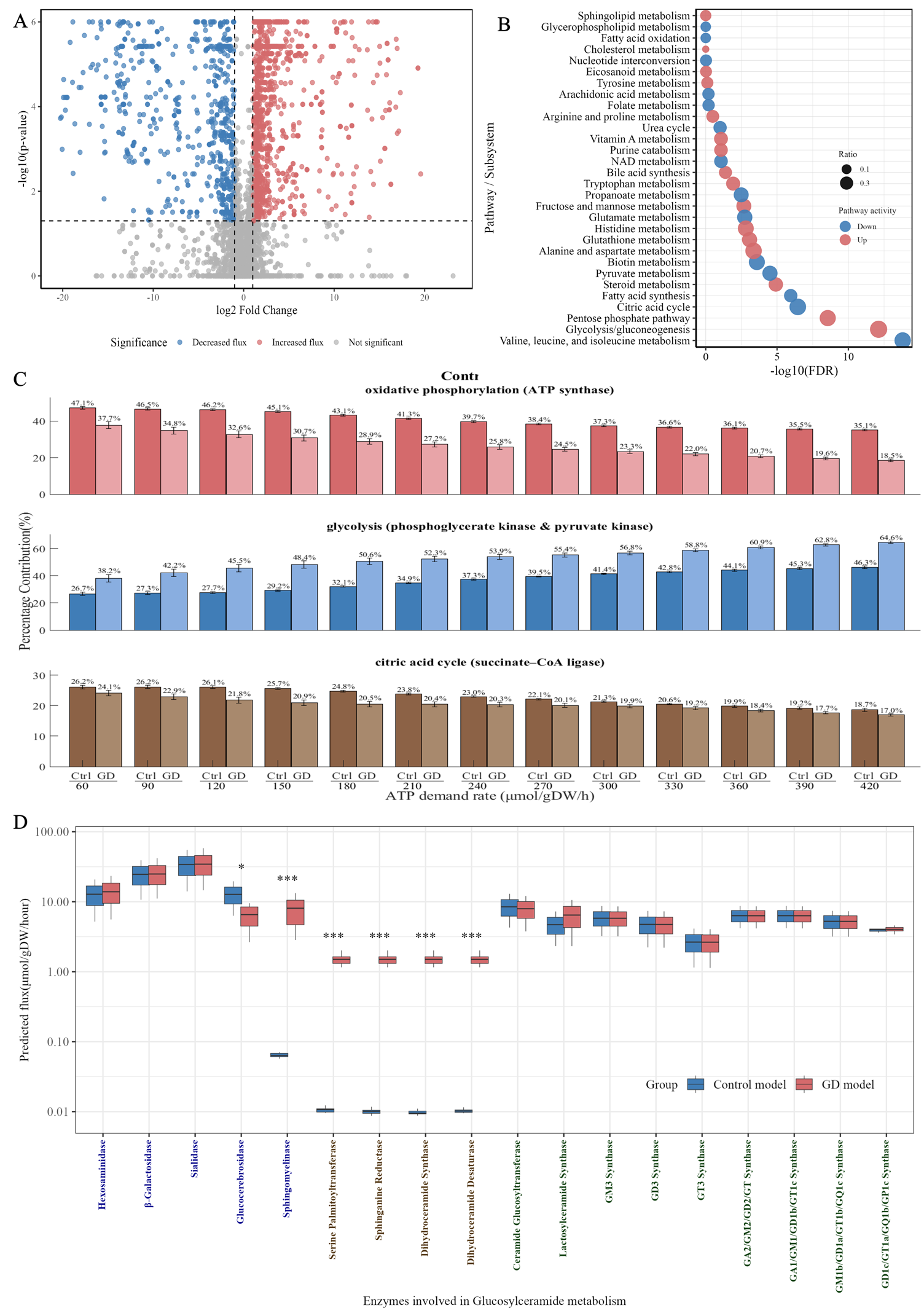


S Figure 6 Impact of Alternative DEG Scaling Factors on Model Predictions (stricter constraints). A stricter constraint was applied to generate GD models (30% % for down-regulated reactions and 170 % for up-regulated reactions). The qualitative metabolic changes highlighted in the main text, particularly at the pathway level, remained consistent across all tested parameter settings. (A) Volcano plot showing fold changes in reaction fluxes between GD and control models as predicted by entropicFBA. (B) Pathway enrichment analysis of reactions with significantly altered fluxes, mapped to metabolic subsystems defined in Recon3D. (C)Predicted ATP contribution from the three main energy-producing pathways. Relative contributions of glycolysis, the citric acid cycle and oxidative phosphorylation to total ATP production in control and GD models, estimated using entropicFBA. (D)Predicted activity of key enzymes in sphingolipid metabolism. Flux distributions for enzymes across the three major branches of sphingolipid metabolism in control and Gaucher disease models are shown on a log_10_ scale (umol/gDW/hour). X-tick labels are colour-coded to indicate pathway assignment: sphingolipid degradation (dark blue), de novo ceramide synthesis (brown) and complex ganglioside synthesis (dark green). Statistical significance is indicated as follows: p < 0.05 (*), p < 0.01 (**), and p < 0.001 (***).
